# How Singing can Help People With Dementia and Their Family Care-Partners: A Mixed Studies Systematic Review With Narrative Synthesis, Thematic Synthesis, and Meta-Integration

**DOI:** 10.3389/fpsyg.2021.764372

**Published:** 2021-10-11

**Authors:** Zara Thompson, Felicity A. Baker, Jeanette Tamplin, Imogen N. Clark

**Affiliations:** Faculty of Fine Arts and Music, University of Melbourne, Melbourne, VIC, Australia

**Keywords:** dementia, family care-partners, singing, choir, music, music therapy, mixed-studies systematic review

## Abstract

**Background:** Recent research on the efficacy of music-based interventions for people with dementia have focused on specific outcomes and methods, and singing has been noted as a particularly beneficial activity. However, due to heterogeneity of research methods, there is a need to synthesise the findings of both quantitative and qualitative research in order to better understand both the impact and potential mechanisms of singing for people in this population.

**Method:** This systematic review included quantitative, qualitative and mixed-methods studies, and analysed these using a systematic mixed-studies synthesis (with a results-based convergent approach). Quantitative and qualitative data were initially synthesised using a narrative synthesis and thematic synthesis method, respectively, before a final meta-integration method was used to synthesise common themes across the two data forms.

**Results:** Electronic and hand search strategies revealed 1,815 relevant studies, 40 of which met the full eligibility criteria. Narrative synthesis of quantitative data revealed six key outcome areas (quality of life; psychological well-being; cognition; engagement; activities of daily living; care-partner well-being), and thematic synthesis of qualitative data generated seven themes relating to the impact and mechanisms of singing (pragmatic elements; social benefits; mood; identity; memory; flow-on effects; and relationships). Meta-integration identified four key areas relating to the impact and mechanisms of singing for people with dementia and care-partners: psychological well-being, quality of life, cognition, and care-partner well-being.

**Conclusion:** Results from the syntheses suggest that singing can positively impact the lives of people with dementia and their care-partners, although due to heterogeneity of study design and outcome measures, it is difficult to draw conclusions based on quantitative data alone. Qualitative data provides further context and insights from participant perspectives, and when integrated with quantitative data, contextual factors that may influence the benefits that participants experience from singing are revealed.

## Introduction

Music is increasingly recognised as a resource for people living with dementia, and in some cases, their family members who support them with informal care. Several recent systematic reviews have synthesised evidence reporting the efficacy of music-based interventions in dementia care (Vasionyte and Madison, 2013; Zhang et al., 2017; van der Steen et al., 2018; Clare and Camic, 2020; Sousa et al., 2020), and although there is significant heterogeneity in the design of music-based programs/interventions, singing is recognised as a prominent method (McDermott et al., 2013). Benefits of singing for health and well-being have been reported for the general population (Daykin et al., 2018), and for people with various mental health or neurological conditions (Williams et al., 2018; Monroe et al., 2020). Several papers have also reported on singing programs specifically for people living with dementia (McCabe et al., 2015; Osman et al., 2016; Unadkat et al., 2016), however no systematic reviews have focused specifically on the efficacy of singing with this population, nor have any explored specifically how singing may be beneficial for people living with dementia and their familial care-partners (henceforth referred to as care-partners).

Meta-analyses of randomised control trials (RCTs) are traditionally considered to be the strongest form of evidence of the efficacy of a health intervention (Evans, 2003). However, methodological challenges in designing research to investigate psychosocial interventions, such as the inability to mask interventions from participants, make RCTs less suitable (Victora et al., 2004). The importance of including the perspectives of people with lived experience of dementia is also gaining recognition, with qualitative research becoming more prominent (Novek and Wilkinson, 2019). It is therefore necessary to examine both quantitative and qualitative research literature to gain a comprehensive understanding of the ways that singing might help people with dementia and their care-partners. For this reason, a mixed-studies approach to systematically reviewing literature has been adopted in this paper.

### Objective/Aim

This paper aims to review the existing literature to explore how singing can support people living with dementia and their care-partners. Sub-questions that guided the synthesis include:

1. What outcomes have been measured in the existing literature?
2. What does the existing literature say about the effectiveness of singing for these outcomes?
3. How do participants describe the experience of being involved in singing interventions/programs?

## Method

This review was registered with Prospero (Centre for Reviews and Dissemination—number CRD42018107628, 11th December 2018) and is reported according to the PRISMA statement (Moher et al., 2009).

### Search Strategy

Searches were conducted across six electronic databases: MEDLINE, EMBASE, PubMed, PsycInfo, CINAHL, and Cochrane Library. Hand searches were also conducted for five journals: Voices: A World Forum for Music Therapy, Australian Journal of Music Therapy, British Journal of Music Therapy, Canadian Journal of Music Therapy, and New Zealand Journal of Music Therapy. Search terms included: dementia, Alzheimer^*^, singing, choir, music, music therapy, and karaoke, with no specific time limit.

### Inclusion/Exclusion Criteria

- Primary focus is on the effects of active singing on people with dementia and/or their familial/informal care-partners (i.e., on the person who is doing the singing)
- Reported in English language
- Published in peer-reviewed journals
- The singing intervention must be clearly described.

Literature was excluded if it featured:

- Case reports, conference papers, personal opinion, and commentary
- Mixed populations (with and without dementia), where results between the groups were not differentiated
- Multiple musical interventions where singing was featured but not the main focus of the intervention, or the percentage of time singing in the program was unclear
- Purely evaluative data with no focus on the effect/impact/experience of the singing on/for the participants
- Studies that featured carer-directed singing (i.e., where a carer sings to a person with dementia to assist them during care routines).

### Selection Process

Search results from each database and hand search were exported into an excel spreadsheet. After duplicates were removed, two reviewers (ZT screened all, FB, JT, and IC assessed one third of results each) independently screened titles and abstracts for eligibility. The full text of articles that appeared eligible based on title/abstract were then reviewed by two independent reviewers. Any discrepancies between reviewer screenings were discussed, and where needed, a third reviewer screened the article for inclusion or exclusion.

### Quality Assessment

Two reviewers independently assessed the quality of each included study (ZT assessed all, FB, JT, and IC assessed one third of results each). The quality checklist by Downs and Black (1998) was used for quantitative studies. Based on previous reporting, we adapted the 27-item checklist (scored out of 32 points), as some items were not relevant to psychosocial interventions, and others were not suitable for non-randomised control studies (NCT) and quasi-experimental studies (McDermott et al., 2013). The total score possible for each type of study was: RCT = 27; NCT = 25; quasi-experimental = 23. For qualitative studies, the Critical Appraisal Skills Program (CASP) was used (Critical Appraisal Skills Programme, 2019), and a combination of the Downs and Black tool and a Mixed-Methods Appraisal Tool (MMAT) was used to evaluate mixed-methods studies (Hong et al., 2018). Results from the quality assessment are presented in Table 1.

**Table 1:** Full results.

**Author (Year) Country**	**Intervention(s) (Length)**	**Context (Facilitator)**	**Sample size**	**Diagnosis (Stage)**	**Participant age (mean)**	**Quality (D&B)**
**RCTs**						
Pongan et al. (2019) France	Group singing Group painting (12 weeks: 2 hr p/w)	Memory clinics (Choir conductor and psychologist)	*n* = 59 (20 male, 39 female)	Probable AD (mild)	Singing = 78.8 Painting = 80.2	74%
Cho (2018) USA	Group singing Group music listening Watching television (4 weeks: 2 × 40 min p/w)	LTCF (Music therapist)	*n* = 52 (43 male, 9 female)	Not reported Stage: Varied	Singing group = 85.06 Listening group = 87.94 TV control group = 87.00	92%
Lyu et al. (2018) China	Group singing Group lyric reading Standard care (3 months: 2 × daily 30–40 min sessions)	Centre for cognitive disorders (Unclear, described as “musicians” and “therapist”)	*n* = 288 (118 male, 170 female)	Probable AD (mild-severe)	Singing group = 68.9 Lyric reading group = 70.3 Control = 69.9	85%
Wang et al. (2018) China	1:1 Singing standard care (3 months: 30–50 mins 3 × per day)	Hospital (unspecified therapist)	*n* = 60 (22 male, 38 female)	AD (mild)	Singing group: 70.4 Control: 69.1	66%
Pongan et al. (2017) France	Group singing Group painting (12 weeks: 2 h p/w)	Memory clinics (Choir conductor and psychologist)	*n* = 59 (20 male, 39 female)	Probable AD (mild)	Singing = 78.8 Painting = 80.2	88%
Särkämö et al. (2016) Finland	Group singing Group music listening Standard care (10 weeks: 1.5 h p/w)	Mixed of community-based (34%) and LTCF (66%) (Singing: Music teacher Listening: Music therapist)	PWD: *n* = 84 (24 male, 60 female) Family CP: *n* = 59 Nurses: *n* = 30	AD/VD/Other (mild)	Singing group = 78.5 Listening group = 79.4 Control = 78.4	74%
Särkämö et al. (2014) Finland	Group singing Group music listening Standard care (10 weeks: 1.5 hr p/w)	Mixed of community-based (34%) and LTCF (66%) (Singing: Music Teacher Listening: Music Therapist)	PWD: *n* = 84 (24 male, 60 female) Family CP: *n* = 59 Nurses: *n* = 30	AD/VD/Other (mild)	Singing group = 78.5 Listening group = 79.4 Control = 78.4	77%
McHugh et al. (2012) USA	Group singing Standard care (3 weeks: ~25 mins × 4 days p/w)	LTCF (Music Therapist)	*n* = 15 (3 male, 12 female)	AD/Related dementia (moderate)	Singing group: 87.5 Control: 86.3	81%
Cooke et al. (2010a) Australia	Group singing Group reading (8 weeks: 3 × 40 min p/w)	LTCF (Musician)	*n* = 47 (14 male, 33 female)	Not reported (moderate)	65–100 years old (82.7% between 75 and 94)	81%
Cooke et al. (2010b) Australia	Group singing Group reading (8 weeks: 3 × 40 min p/w)	LTCF (Musician)	*n* = 47 (14 male, 33 female)	Not reported (moderate)	65–100 years old (82.7% between 75 and 94)	85%
Harrison et al. (2010) Australia	Group singing Group reading (8 weeks: 3 × 40 min p/w)	LTCF (Musician)	*n* = 47 (14 male, 33 female)	Not reported (moderate)	65–100 years old (82.7% between 75 and 94)	77%
**NCTs**						
Chen et al. (2019) China	Group singing (Chinese Opera) Standard care (12 weeks: 2 × 40 min p/w)	LTCF (Musicians and Researchers)	*n* = 43 (11 male, 32 female)	Not reported (mild-moderate)	Singing group = 83 Control = 85	80%
Satoh et al. (2015) Japan	Group singing and at-home Karaoke Standard care (25 weeks:1 h p/w)	Community based (Musicians)	*n* = 20 (6 male, 14 female)	Probable AD (mild-moderate)	Singing group = 78.1 Control = 77.0	64%
Takahashi and Matsushita (2006) Japan	Group singing Standard care (2 years: 1 h p/w)	LTCF (Music Therapists)	*n* = 43 (10 male, 33 female)	CD, VD, AD, Parkinson's Type (moderate-severe)	Singing: 82.7 Non-random control: 84.9	68%
**Quasi-experimental designs**						
Maguire (2021) USA	1:1 Singing (5 weeks: 40–50 min p/w)	LTCF (Musicians)	*n* = 25 (female)	Not reported (not reported)	Not reported	47%
Hiller (2020) USA	Group singing (4 weeks: 3 × approx. 30 min per fortnight)	LTCF (Music therapist)	*n* = 28 (7 male, 21 female)	AD and Related dementias (mild-severe)	Range: 63–99	73%
Fraile et al. (2019) France	1:1 Singing (5 weeks: 2 × 20 min p/w)	Mix of LTCF and Community-based (Speech Pathology Students)	*n* = 12 (5 male, 7 female)	AD (mild-moderate)	83.83	68%
de la Rubia Orti et al. (2018) Spain	Group singing (unclear—appears to be single-session)	LTCF (Music therapist)	*n* = 25 (22.73% male, 77.27% female)	AD (mild)	78.38	78%
Moussard et al. (2014) Canada	1:1 Singing 1:1 Poetry (6 weeks: 45 min p/w)	Community based (unclear)	*n* = 15 (6 male, 9 female)	AD (mild)	77.8	57%
Lesta and Petocz (2006) Australia	Group singing (4 days: 30 min per session)	LTCF (Music therapist)	*n* = 4 (female)	Not reported (moderate)	91.25	70%
Groene et al. (1998) USA	Group singing Group exercise (3 weeks: 13–16 sessions in total, 20–45 min per session)	Community-based (Music therapist/Occupational therapist)	*n* = 7 (1 male, 6 female)	AD (moderate-severe)	85.3	47%
Korb (1997) USA	Group singing Group rhythm Group discussion 12 weeks (2 × 30 min p/w; 8 sessions per intervention in total)	Community-based (Music therapist)	*n* = 9 (male)	Dementia or AD (not reported)	Not reported	60%
Hanson et al. (1996) USA	Group singing Group rhythm Group movement (12 weeks: 2 × 30 min p/w)	Mix of LTCF, Community-based and hospital (Music therapist)	*n* = 51 (10 male, 41 female)	AD and Related dementias (mild-severe)	82	78%
Prickett and Moore (1991) USA	1:1 Singing 1:1 Speaking (3 weeks: 20 min p/w)	Hospital (Recreational therapist)	*n* = 10 (4 male, 6 female)	Probable AD (not reported)	75	57%
Clair and Bernstein (1990) USA	1:1 Singing 1:1 Drumming (14 weeks: 10 min p/w)	LTCF (Music therapist)	*n* = 6 (male)	AD (severe)	Range 62–73	57%
Olderog Millard and Smith (1989) USA	Group singing Group discussion (5 weeks: 2 × 30 min p/w)	LTCF (unclear)	*n* = 10 (3 male, 7 female)	AD, OBS (moderate)	81.4	57%
Mittelman and Papayannopoulou (2018) USA	Group singing (12 weeks: 2 h p/w)	Community-based (Musician, with input from Music therapist)	PWD: *n* = 11 (7 male, 4 female) CP: *n* = 11 (5 male, 6 female)	Not Reported (not reported)	PWD = 79.4 CP = 71.7	41%
Tamplin et al. (2018) Australia	Group singing (20 weeks: 2 h p/w)	Community-based (Music therapists)	PWD: *n* = 9 (4 male, 5 female) CP: *n* = 9 (4 male, 6 female)	Not reported (mild-moderate)	PWD = 77.9 CP = 73.0	64%
Davidson and Almeida (2014) Australia	Group singing (6 weeks: 2 r p/w)	Mix of LTCF and Community-based (Musician)	*N =* 12 (6 PWD, 6 CP)	Not reported (mild-moderate)	PWD = 79.50 CP = 69.67	41%
Camic et al. (2011) UK	Group singing (10 weeks: 90 min p/w)	Community-based (Musician)	PWD: *n* = 10 (5 male, 5 female) CP: *n* = 10 (4 male, 6 female)	AD, VD, Mixed Dementia, MCI (mild-severe)	PWD = 75 CP = not reported	70%
Davidson and Fedele (2011) Australia	Group singing (6 weeks: 2 h p/w)	Mix of LTCF and Community-based (Musician)	PWD: *n* = 27 (9 male, 18 female) CP: *n* = 19 (4 male, 15 female)	Not reported (mild-moderate)	PWD = 82.67 CP = 57.91	41%
**Qualitative studies**						
**Author (Year) Country**	**Intervention (Length)**	**Context (Facilitator)**	**Sample size**	**Diagnosis (Stage)**	**Participant age (mean)**	**Quality (CASP)**
Lee S. et al. (2020) Ireland	Group singing (6 weeks: 1 h p/w)	Community-based (Music therapist)	PWD: *n* = 3 (male) CP: *n* = 4 (1 male, 3 female)	Not reported (mild)	PWD: Range = 70–89 CP: Range = 30–79	100%
Clark et al. (2018) Australia	Group singing (20 weeks: 2 h p/w)	Community-based (Music therapists)	PWD: *n* = 10 (5 male, 5 female) CP: *n* = 10 (4 male, 6 female)	Not reported (mild-moderate)	PWD = 79.1 CP = 75.7	90%
Harris and Caporella (2018) USA	Group singing (8 weeks: 90 min. p/w, plus performance)	Community-based (Musicians)	PWD: *n* = 6 (3 male, 3 female) CP: *n* = 7 (3 male, 4 female) College students: *n* = 13 (all female)	AD, MCI (mild)	PWD = 72 CP = 65 Students = 20.5	60%
Osman et al. (2016) UK	Group singing (unclear, participants attended minimum of 2 sessions)	Community-based (Musicians)	PWD: *n* = 10 (5 male, 5 female) CP: *n* = 10 (2 male, 8 female)	Not reported (not reported)	Not reported	65%
Unadkat et al. (2016) UK	Group singing (unclear, range of different groups and lengths included)	Community-based (Musicians)	PWD: *n* = 17 (9 male, 8 female) CP: *n* = 17 (8 male, 9 female)	AD, VD, Mixed Dementia, FTD, Cadasil, Unspecified (mild-severe)	PWD = 72.2 CP = 70.3	95%
McCabe et al. (2015) UK	Group opera program (~1 year)	Community-based (Musicians)	Not specified	Not reported (not reported)	Not reported	75%
Dassa and Amir (2014) Israel	Group singing (1 month: 2 × 45 min. p/w)	LTCF (Music therapist)	PWD: *n* = 6 (2 male, 4 female)	AD (moderate-severe)	78.5	80%
Harris and Caporella (2014) USA	Group singing (10 weeks: 90 min. p/w, plus performance)	Community-based (Musicians)	PWD: *n* = 22 (9 male, 13 female) CP: *n* = 21 (7 male, 14 female)	AD, MCI, PPA, LB (mild)	PWD = 72.5 CP = 72.3 Students = 19.8	80%
Hara (2011) UK	Group singing (2 years, weekly)	Community-based (Musicians)	Not specified (due to nature of ethnographic design)	Not reported (moderate-severe)	unclear (participants in case example described as in their 70 and 80s)	60%

*RCT, Randomised Control Trial; NCT, Non-Randomised Control Trial; LTCF, Long Term Care Facility; PWD, people with dementia; CP, care partners; AD, Alzheimer's disease; VD, Vascular Dementia; CD, Cerebrovascular Dementia; MCI, Mild Cognitive Impairment; PPA, Primary Progressive Aphasia, LB, Dementia with Lewy Bodies; FTD, Fronto-Temporal Dementia; TV, Television*.

### Data Extraction

The first author extracted data from each study into an excel spreadsheet using a standard data extraction form, which included study design, data source, participant demographics, length, location and type of intervention, study objectives, and outcomes.

### Data Synthesis

**Figure 1:**
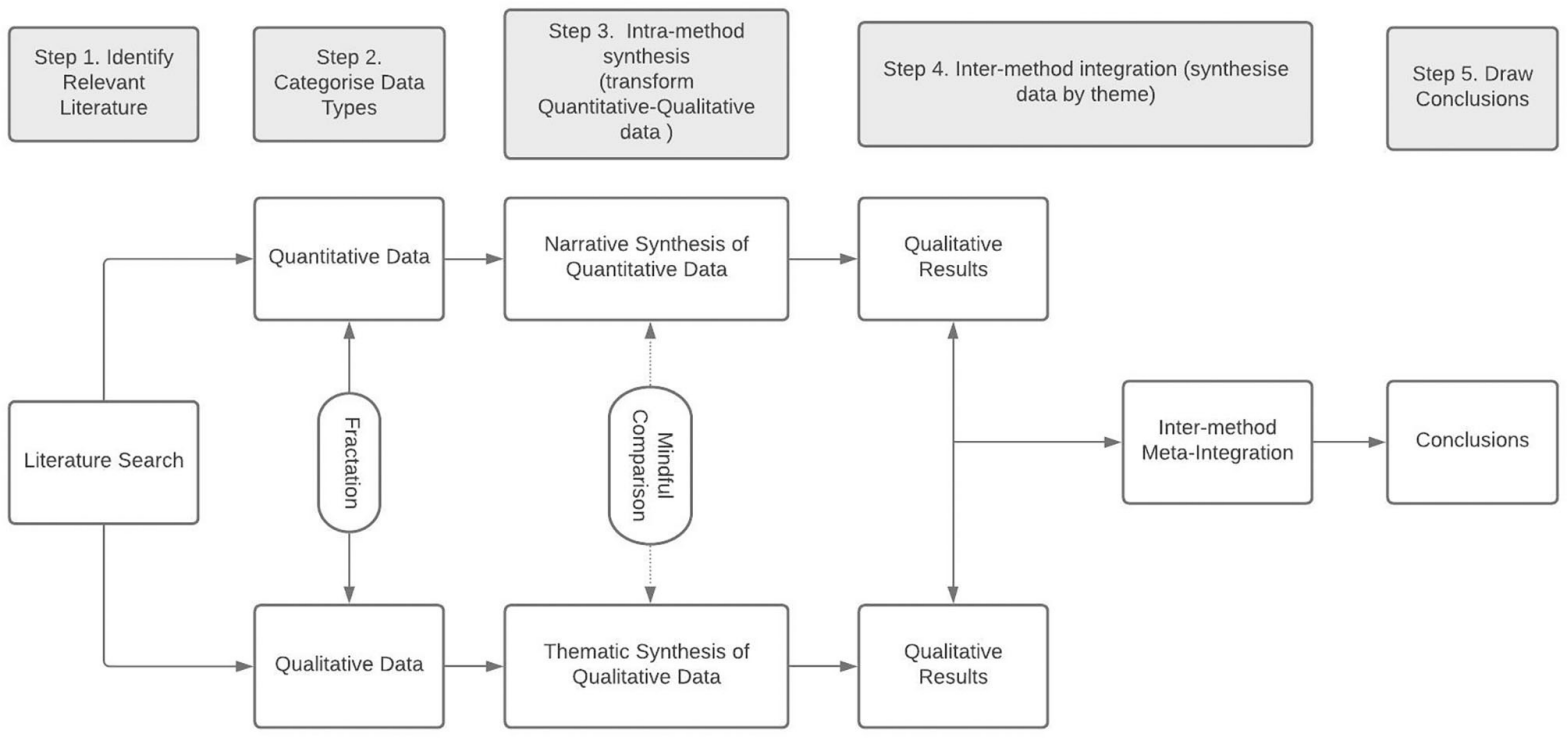
Steps for mixed studies synthesis, adapted from Frantzen and Fetters (2016) and Hong et al. (2017).

As this review included heterogenous quantitative, qualitative and mixed method studies, a systematic mixed-studies synthesis results-based convergent approach was selected (Pluye and Hong, 2014; Hong et al., 2017). Quantitative and qualitative data were synthesised separately, and then brought together in a final synthesis (Figure 1). A narrative synthesis approach (Popay et al., 2006) was performed whereby quantitative data was translated into words for synthesis with qualitative data (Frantzen and Fetters, 2016). Qualitative data was thematically synthesised (Thomas and Harden, 2008) followed by a meta-integration (Frantzen and Fetters, 2016) to merge the quantitative and qualitative data in a final synthesis. Brief descriptions for each stage of synthesis are presented below.

#### Synthesis of Quantitative Data—Narrative Synthesis

**Figure 2:**
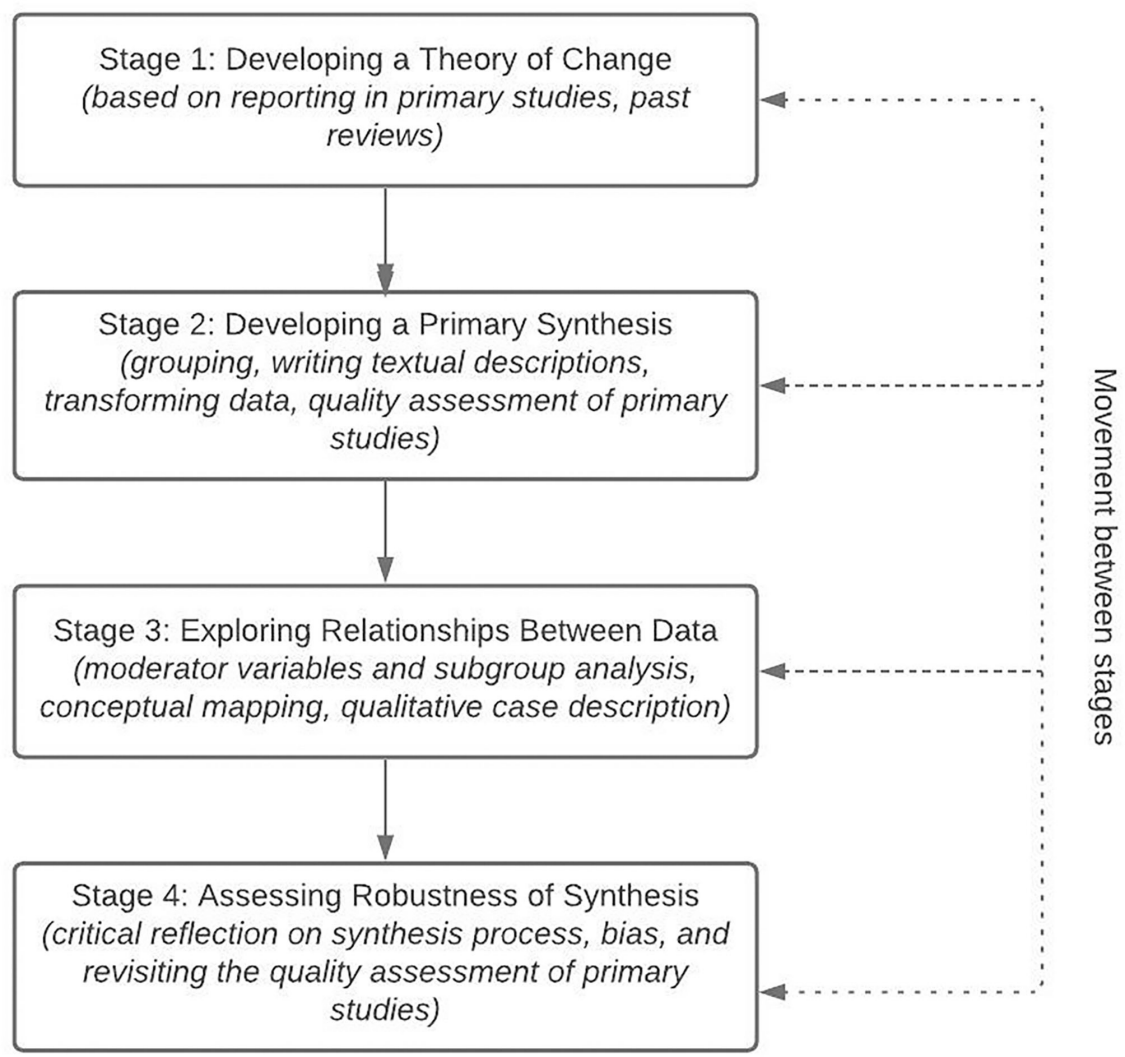
Process of narrative synthesis of quantitative data, adapted from Popay et al. (2006).

Four iterative stages (Figure 2) characterised the narrative synthesis process (i) theory development; (ii) preliminary synthesis; (iii) exploring relationships between studies; and (iv) assessment of robustness (Popay et al., 2006).

#### Synthesis of Qualitative Data—Thematic Synthesis

**Figure 3:**
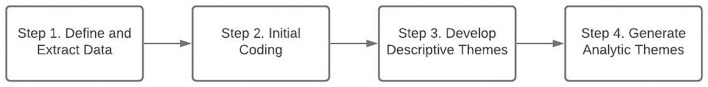
Thematic synthesis process, adapted from Thomas and Harden (2008).

A four-step thematic synthesis process adapted from Thomas and Harden (2008) was used to synthesise qualitative data (Figure 3). Findings sections of each paper were imported into a MaxQDA file (MAXQDA, 2020) (VERBI Software, 2019), where initial codes and descriptive themes were developed (steps 2–3, Thomas and Harden, 2008). Inductive coding and thematic development were adopted to avoid overlooking any novel findings due to a priori assumptions. Once the descriptive themes were developed, Author 1 returned to the research question and explored the relationships between themes to generate the final analytic themes.

#### Synthesis of All Data—Meta-Integration

Meta-integration was undertaken, whereby the results from the independent quantitative and qualitative syntheses were brought together in a process of “cross-checking, connecting and co-informing” (Frantzen and Fetters, 2016, p. 2267). Synthesis techniques were also used to make sense of the data, including: exploring moderator variables (asking “who,” “where,” and “why” of the data); developing conceptual maps (comparing and contrasting findings); and triangulation based on how the data was produced (Popay et al., 2006).

## Results

**Figure 4:**
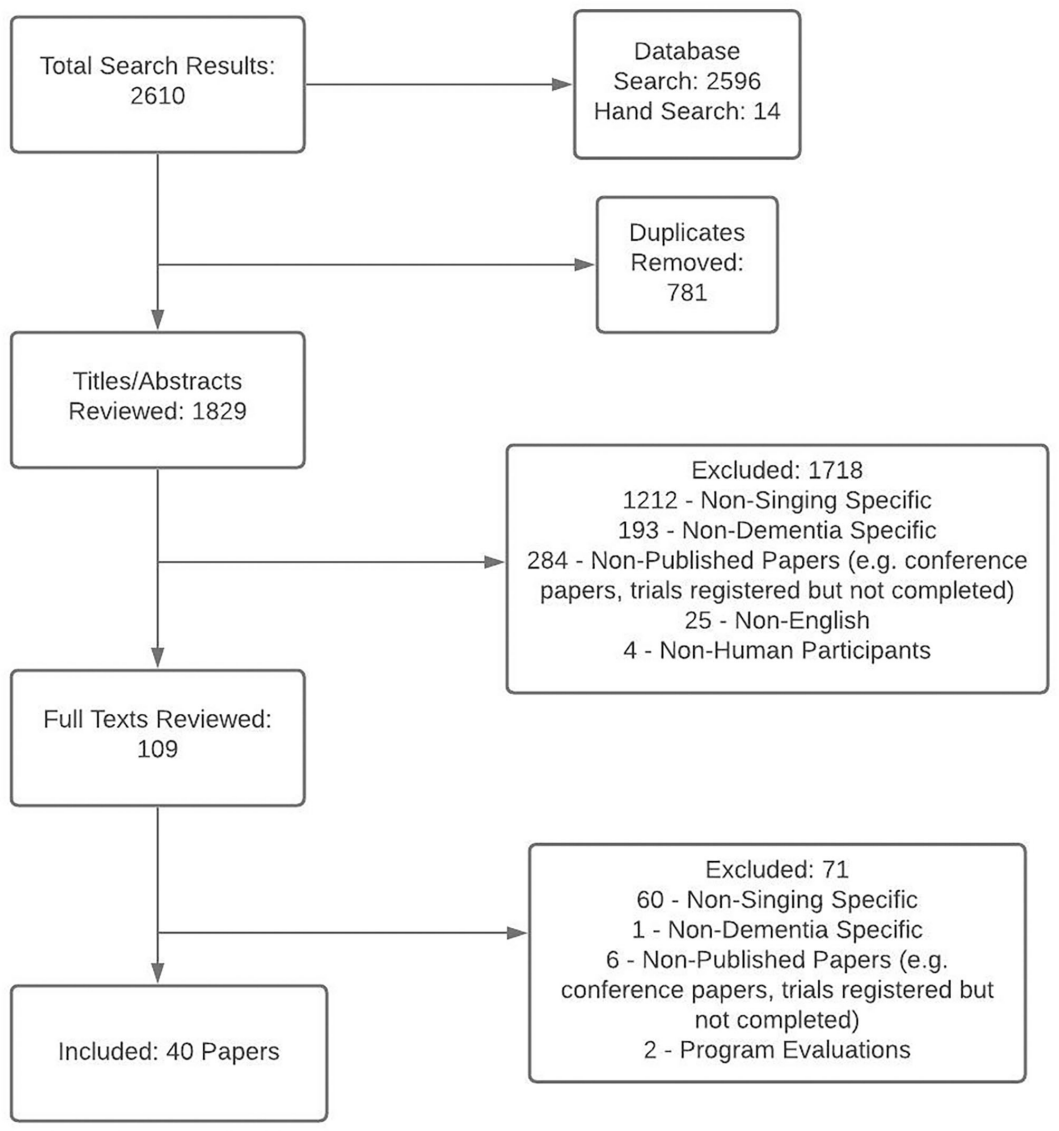
PRISMA Flowchart depicting study selection process.

The electronic search of databases (January 2021) yielded 1,815 unique results, with an additional 14 papers identified through hand searches. Of these, 1,718 papers were excluded based on a review of titles/abstracts, and an additional 71 papers were excluded following a review of the full-text articles. There were 40 papers that met all inclusion criteria: 26 quantitative, 9 qualitative, and 5 mixed-method papers. Three studies were reported in two papers (Särkämö et al., 2014, 2016; Pongan et al., 2017, 2019; Clark et al., 2018; Tamplin et al., 2018), and a further study by Cooke et al. was reported in three papers (Cooke et al., 2010a,b; Harrison et al., 2010). Figure 4 depicts the study selection process. Full results are presented in Table 1. Demographic data regarding the context and types of interventions in the included papers is summarised in Table 2.

**Table 2:** Demographic outcomes of included studies.

**Country of study**	
Australia	8
Canada	1
China	3
Finland	2
France	3
Ireland	1
Israel	1
Japan	2
Spain	1
UK	5
USA	13
**Context of intervention**	
Long Term Care Facility (LTCF)	14
Community-based	15
Mix (LTCF and Community)	6
Hospital	2
Unclear	3
**Facilitator**	
Musician	20
Music therapist	13
Speech pathologist	1
Unclear	6
**Type of intervention**	
Group singing	34
Individualised (1:1) Singing	6

### Mixed Method Studies

Five studies used a mix of qualitative and quantitative measures (Camic et al., 2011; Davidson and Fedele, 2011; Davidson and Almeida, 2014; Mittelman and Papayannopoulou, 2018; Tamplin et al., 2018). As none of these studies integrated their qualitative and quantitative data, their results were separated out in a process of fractionation (Frantzen and Fetters, 2016) and the relevant data from each were included with the quantitative and qualitative syntheses, respectively.

### Narrative Synthesis of Quantitative Data

Data from quantitative and mixed method studies were extracted into a table and grouped according to the types of outcomes measured. Six major outcome categories were identified: Quality of Life (QOL), Psychological Well-being, Cognition, Engagement, Activities of Daily Living and Care-Partner Outcomes. The results and discussion for each outcome-category are presented below.

#### Quality of Life

Nine included studies measured QOL, all of which featured group-singing interventions (Table 3). Three studies reported a significant improvement in overall QOL based on self-report measures only (Pongan et al., 2017; Cho, 2018; Mittelman and Papayannopoulou, 2018). Cho (2018) reported a significant improvement in QOL following group singing, compared to a music listening intervention and television-watching control. A pre-post-test study also found improvements in two different measures of QOL following group singing (Mittelman and Papayannopoulou, 2018). However, the authors opted to report significance at *p* ≤ 0.1 owing to small sample size (*n* = 10), so these results should be cautiously interpreted. Pongan et al. (2017) reported significant improvements in QOL following both group singing and group painting interventions. Cooke et al. (2010b) observed a similar phenomenon upon sub-analysis; they reported that for participants who attended at least 50% of their intervention, their score on the “self-esteem” item on the QOL measure improved significantly, regardless of intervention (group singing or reading group). The authors in each of these studies speculate that the improvement in QOL evident in both types of interventions may have been due to the introduction of regular social activities for participants, rather than the nature of the activities themselves.

**Table 3:** Overview of results for quality of life outcomes.

**Study (Year)**	**Design**	**Outcome measure**	**Results Reported by Authors (*n* = participants per group)**	**Quality (D&B)**
Chen et al. (2019)	RCT	QOL-AD	No total score between group differences, between group improvement for singing group (*n* = 21) on three items on QOL-AD measure (emotions, compatibility with friends, entertainment capability)	80%
Cho (2018)	RCT	QOL-AD	Within group improvement for singing (*n* = 14)	92%
Pongan et al. (2017)	RCT	EQ-5D	Within group improvements for painting (*n* = 28) and singing (*n* = 31)	88%
Särkämö et al. (2014); Särkämö et al. (2016)	RCT	QOL-AD (self and proxy)	No between group improvements Within group improvement for music listening (*n* = 29), no change for singing (*n* = 30)	77%
Cooke et al. (2010b)	RCT	DQOL	No within group changes for either group Between group improvement on DQOL item (sense of belonging) at mid-point for reading (*n* = 21) compared with singing (*n* = 23)	85%
Maguire (2021)	QE	SWLS	Between group decrease (worsening) in singing group (*n* = 7) compared to control (*n* = 12)	47%
Tamplin et al. (2018)	QE	QOL-AD (self and proxy)	No pre-post change Significant difference between self-reported and proxy scores (*n* = 9)	78%
Mittelman and Papayannopoulou (2018)	QE	QOL-AD DemQOL (self and proxy)	Pre-post improvement on both measures (*n* = 10)	69%
Davidson and Fedele (2011)	QE	QOL-AD (self and proxy)	No total score pre-post change (*n* = 27) Improvement for one item in proxy measure (living situation)	41%
Camic et al. (2011)	QE	DemQOL-4 (self and proxy)	No pre-post change (*n* = 10)	70%

*RCT, Randomised Control Trial; QE, Quasi-Experimental Design; Self, self report measure; Proxy, proxy report measure; QOL-AD, Quality of Life in Alzheimer's Disease; EQ-5D, EuroQol Five-Dimensional; DQOL, Dementia Quality of Life Instrument; DemQOL, Dementia Quality of Life; SWLS, Satisfaction with Life Scale*.

Of the five studies that reported no significant change, three conducted a sub-analysis and reported significance on particular items on the QOL measures (Cooke et al., 2010b; Davidson and Fedele, 2011; Chen et al., 2019). In addition to significant improvements in self-esteem, Cooke et al. (2010b) observed that participants in a “reading” control group reported significantly higher sense of belonging than those in the singing group. The authors suggested this may have been due to differences in facilitation styles, as the singing groups were more structured, with less opportunities for organic discussion than the reading groups. In a pre-post study, Davidson and Fedele (2011) reported a significant improvement on the proxy-rated “living situation” item on the QOL-AD, suggesting that carer perspectives on the living situation of participants with dementia improved following group singing. Another pre-post study by Chen et al. (2019) reported significant improvements in QOL domains measuring friendships, mood and ability to experience enjoyment. Three studies that reported high baseline QOL and no significant change, indicating possible ceiling effects with relatively good QOL prior to the interventions and no deterioration throughout the project (Cooke et al., 2010b; Camic et al., 2011; Tamplin et al., 2018).

Across the four RCTs that measured QOL, the results were varied, and the study designs and interventions were heterogenous (Table 3). Cho (2018) reflected several differences between the treatment and control interventions that may have given their group singing intervention an advantage over the controls; training and experience of the facilitator, the types of interventions, and types and levels of engagement demanded of participants. Cooke et al. (2010b) similarly reflected that the less-structured format of their reading control group may have fostered more opportunities for connexion than their structured singing group, which may account for the difference in this score. Särkämö et al. (2014) suggested that the significant improvement noted in their music-listening control group may have been due to the ease of care-partners being able to implement techniques learnt from the music-listening intervention at home, therefore having a longer-term effect on QOL.

#### Psychological Well-Being

Several included studies measured outcomes relating to different aspects of psychological well-being, including depression, anxiety, agitation, and neuropsychiatric outcomes (Table 4). Historically, these types of outcomes have been classified in the dementia literature as “behavioural and psychological symptoms of dementia” or “BPSD.” However, many academics and advocates are calling for a change in terminology around BPSD due to stigma, lack of acknowledgement of other potential causes that may trigger such “symptoms” (such as inadequate environment and/or support), and reliance on imperfect pharmacological treatments (Madhusoodanan et al., 2007; Swaffer, 2015; Macaulay, 2018). With this in mind, we have chosen to use the term “psychological well-being” to describe the aforementioned outcomes that were featured in the studies included in this review. Results for each outcome-category follow.

**Table 4:** Overview of results of psychological wellbeing outcomes.

**Study**	**Design**	**outcome measure**	**Reported results**	**Quality (D&B)**
**Neuropsychiatric Inventory**				
Wang et al. (2018)	RCT	NPI	Between group improvement for singing (*n* = 30) compared to control Within group improvement for singing group (*n* = 30)	66%
Lyu et al. (2018)	RCT	NPI	Between group improvements at immediate follow for singing (*n* = 97) and lyric reading (*n* = 96) compared to control (*n* = 95) Between group improvement for singing group at 3-month follow up compared with both reading and control	85%
Chen et al. (2019)	NCT	CNPI	Between group improvements on five domains of CNPI for singing (*n* = 21): depression, anxiety, irritability, aberrant motor behaviour, and eating disorders compared to control (*n* = 22)	80%
Satoh et al. (2015)	NCT	NPI	Within group improvement for singing only (*n* = 10)	64%
Camic et al. (2011)	QE	NPI	No significant change (*n* = 10)	70%
**Agitation**				
Cooke et al. (2010a)	RCT	CMAI-SF	No significant change—mean scores suggested consistently low instances of agitation across sample (floor effect) (*n* = 47)	85%
Tamplin et al. (2018)	QE	CMAI-SF	No significant change– mean scores suggested consistently low instances of agitation (floor effect) (*n* = 47)	78%
**Anxiety**				
Pongan et al. (2017)	RCT	STAI	Within group improvement for both singing (*n* = 31) and painting (*n* = 28), with greater effect size for painting	88%
Cooke et al. (2010a)	RCT	RAID	No significant change—mean scores indicated consistently low levels of anxiety (floor effect) (*n* = 47)	85%
Tamplin et al. (2018)	QE	RAID	No pre-post change (*n* = 9)	78%
de la Rubia Orti et al. (2018)	QE	HADS	Pre-post improvement (*n* = 25), inversely corelated with decrease in cortisol	78%
**Depression**				
Pongan et al. (2017)	RCT	GDS	Between group improvement for painting (*n* = 28) compared to singing (*n* = 31)	88%
Särkämö et al. (2014)	RCT	CBS	Between group improvements for both groups (singing (*n* = 27) and music listening (*n* = 29) compared to standard care control group (*n* = 28)	77%
Cooke et al. (2010b)	RCT	GDS	No significant change—mean scores indicated consistently low levels of depression (floor effect) (*n* = 47)	81%
Tamplin et al. (2018)	QE	AES (self and proxy)	No pre-post change (*n* = 9) Significant difference between self and proxy report scores at baseline and post intervention	78%
de la Rubia Orti et al. (2018)	QE	HADS	Pre-post decrease (improvement) correlated with decreased cortisol levels (*n* = 25)	78%
Camic et al. (2011)	QE	GDS	Pre-post increase (worsening) (*n* = 10)	70%
**Immediate well-being**				
Pongan et al. (2019)	RCT	EVIBE	Within group improvement for both singing (*n* = 31) and painting (*n* = 28) groups	74%
Lesta and Petocz (2006)	QE	MBAC	Pre-post improvement in mood scale (*n* = 4)	69%

*RCT, Randomised Control Trial; NCT, Non-Randomised Control Trial; QE, Quasi-Experimental Design; NPI, Neuropsychiatric Inventory; CNPI, Chinese Neuropsychiatric Inventory; CMAI-SF, Cohen-Mansfield Agitation Inventory- Short Form; STAI, State-Trait Anxiety Inventory; RAID, Rating Anxiety in Dementia; CBS, The Cornell-Brown Scale for Quality of Life in Dementia; GDS, Geriatric Depression Scale; AES, Apathy Evaluation Scale; HADS, Hospital Anxiety and Depression Scale; EVIBE, Evaluation Instantane e du Bien-Etre; MBAC, Mood-Behaviour Assessment Chart*.

##### Neuropsychiatric Outcomes

Five studies used the Neuropsychiatric Inventory (NPI) to measure the impact of singing on changes in mood and behaviour for people with dementia. The NPI measures change across a range of domains: depression, anxiety, elation, irritability, disinhibition and apathy, delusions, hallucinations, agitation, motor disturbances, and changes to eating and sleeping patterns (Cummings, 2020). Three studies reported significant reduction in total NPI score following a group singing intervention (2 RCTs, one NCT) (Satoh et al., 2015; Lyu et al., 2018; Wang et al., 2018). Chen et al. (2019) used a translated version of the NPI (C-NPI), and while they did not report global/total improvement, there was significance for C-NPI domains measuring depression, anxiety, irritability, repetitive movements, and disordered eating. One study reported no significant improvement in NPI scores, however, the authors noted floor effects, suggesting participants were not experiencing these challenges at baseline (Camic et al., 2011). Intervention dosage may also have impacted results; the three studies that reported significant improvements had either more frequent sessions, or the intervention period lasted longer than the study that found no significant results (Table 4).

##### Agitation

Two studies used the Cohen-Mansfield Agitation Inventory (CMAI) to measure agitation, both of which reported no statistically significant changes in score, likely due to a floor effect (Cooke et al., 2010a; Tamplin et al., 2018). Tamplin et al. (2018) theorised that this may have been indicative of selection bias, as it is possible that the type of people who would volunteer to join their community-based program may not be experiencing agitation prior to joining. Conversely, however, participants in the study by Cooke et al. (2010a) were screened based on recent clinical reports of agitation by staff at the care facility where participants resided, and still yielded a low baseline score. The authors speculated that this may indicate a discrepancy between how staff report agitation and what formal measures of agitation capture.

##### Anxiety

Four studies measured the effect of group singing on anxiety. Pongan et al. (2017) reported significant within group reductions in anxiety for both the singing intervention and active control (painting), with a significant between group reduction favouring the painting intervention. Tamplin et al. (2018) and Cooke et al. (2010a) reported no significant reduction in anxiety following their respective group singing interventions. Cooke et al. (2010a) reported that this was likely due to a floor effect. However, Tamplin et al. (2018) observed a small, non-significant effect (*d* = 0.28*)* suggesting decreased anxiety scores, which they reported as clinically significant given the small sample size. de la Rubia Orti et al. (2018) reported a significant decrease in anxiety, but that this was inversely correlated with a decrease in cortisol levels, which occurred during singing.

##### Depression

Six studies measured the impact of singing on depression (Cooke et al., 2010b; Camic et al., 2011; Särkämö et al., 2014; Pongan et al., 2017; de la Rubia Orti et al., 2018; Tamplin et al., 2018). Three studies used the Geriatric Depression Scale (Cooke et al., 2010a; Camic et al., 2011; Pongan et al., 2017), and one used the Hospital Anxiety and Depression Scale (de la Rubia Orti et al., 2018). Särkämö et al. (2014) used the Cornell-Brown Scale (CBS) for QOL, which is a modified form of the CBS scale for Depression for people with dementia (Ready et al., 2002). The CBS-QOL includes domains relating to depressive symptoms such as mood, ideation, behavioural, physical and functional signs of depression, suggesting that this can also assess depression (Ready et al., 2002). Särkämö et al. (2014) used this to assess changes in depressive symptoms; therefore, we have chosen to include the CBS-QOL here, rather than the QOL section.

In their medium-quality pre-post study, de la Rubia Orti et al. (2018) found that depression scores significantly reduced following a singing intervention, correlating with an observed reduction in cortisol levels. Särkämö et al. (2014) reported short-term within group reductions in depressive symptoms following both group singing and music listening, however, these changes were not maintained at the 3-month follow up. The authors hypothesise that regular sessions are needed to maintain the positive effects on depression. Although Cooke et al. (2010b) found no significant improvement in depression scores initially, they attributed this to floor effects. However, on sub-group analysis of participants with higher scores at baseline (*n* = 12), they found significant decreases in depression for people in both the singing and reading groups. As per QOL outcomes, program regularity may be more important for improving depression symptoms than specific activities.

Conversely, Pongan et al. (2017) found that depression was only reduced for participants in the painting control group. The authors theorised that this may have been due to differences in the way that the sessions were facilitated; painting was more introspective and creative, whereas the singing groups were more structured and demanded more of participants socially and emotionally.

Camic et al. (2011), reported a significant increase in depression scores following their weekly singing group program, but noted that this may be expected in the context of participants with dementia as their symptoms progress. Tamplin et al. (2018) also reported a similar expectation of increasing depression in the dementia trajectory and found no improvements in apathy in their study (however, there was a ceiling effect for apathy). The evidence from these two studies is weak due to the small sample-sizes and pre-post design, however, the observations may be clinically important.

##### Immediate Well-Being

One fair quality study compared the effects of group singing and group painting on an immediate sense of well-being (Pongan et al., 2019). They found that participants in both groups reported improved well-being immediately following the sessions, which aligns with the findings from their previous study (Pongan et al., 2017). One further (medium quality) study (Lesta and Petocz, 2006) used a bespoke tool to measure mood, non-social, and social behaviour for participants who were reportedly experiencing “Sundowner's Syndrome.” This study found mostly significant improvements across the domains during the singing intervention, and in the 15 min following the sessions. The results of this study should be interpreted with caution, however, due to the non-standardised measure and small sample size.

#### Cognition

Cognition was the most common outcome included in the quantitative papers (14 studies) (Table 5). However, the measures and constructs were heterogenous across included studies, which prohibited meta-analysis. This section will discuss three broad ways that cognition was investigated: cognitive screening tools, neuropsychological batteries, and testing specific memory training interventions.

**Table 5:** Overview of results for cognitive outcomes.

**Study**	**Design**	**Outcome measure**	**Results**	**Quality (D&B)**
**Cognitive screening tools**				
Wang et al. (2018)	RCT	MMSE MoCA	MMSE: Between group improvement for singing (*n* = 30) compared to control (*n* = 30) MoCA: Between group improvement for singing (*n* = 30) compared to control (*n* = 30), within group improvement for singing at immediate and 3-month follow-up	66%
Cooke et al. (2010a)	RCT	MMSE	No significant change (*n* = 47)	85%
Chen et al. (2019)	NCT	MMSE	No between group improvement in total score, between group improvement for singing group (*n* = 21) on “recall” subscale compared to control (*n* = 22)	80%
Takahashi and Matsushita (2006)	NCT	R-HDS	No significant change (*n* = 43)	
Maguire (2021)	QE	MMSE (& R-MMSE) CDT (& R-CDT) NARR CS	MMSE & R-MMSE: no significant change CDT: Between group improvement for singing group (*n* = 7) compared to control (*n* = 15) R-CDT: Between group improvement for singing group (*n* = 7) compared to control (*n* = 12) NAAR: no significant change CS: Between group improvement for singing group (*n* = 8) compared to control (*n* = 5)	47%
Camic et al. (2011)	QE	MMSE ACE-R	No pre-post change on either measure (*n* = 10)	70%
Davidson and Fedele (2011)	QE	HDS	No significant change (*n* = 27)	41%
**Neuropsychological batteries**				
Lyu et al. (2018)	RCT	MMSE WHO-UCLA AVLT SVFT	MMSE: no significant change (*n* = 288) WHO-UCLA AVLT: no significant change (*n* = 288) SFVT: between group improvement for singing (*n* = 97) and music listening (*n* = 96) compared to control (*n* = 95) at immediate follow up; between group improvement for singing (*n* = 97) compared to control (*n* = 95) at 6 month follow up	85%
Pongan et al. (2017)	RCT	Neuropsychological Battery: FCRT - TMT - DST - Digit Span -Stroop Test - LCFT -FAB	FCRT: no overall change, between group decrease (worsening) for painting (*n* = 28), compared to singing (*n* = 31) on one item (total recall) Stroop Test: Within group improvements for both groups (decreased interference errors), non-significant trend to greater improvement in singing group (*n* = 31) No significant results for other tests	88%
Särkämö et al. (2014)	RCT	Neuropsychological Battery: - General cognition - Orientation - Short-term and working memory - Verbal learning - Delayed memory - Verbal skills - Visuospatial skills - Attention and executive function	Immediate follow up: General cognition: between group improvement for singing (*n* = 27) and music listening (*n* = 29) compared to control (*n* = 28) Attention and executive function: between group improvement for singing (*n* = 27) and music listening (*n* = 29) compared to control (*n* = 28) Short term and working memory: between group improvement for singing (*n* = 27) compared to music listening (*n* = 29) and control (*n* = 28) Long term (9 month) follow up: Orientation: Between group decline (worsening) for control (*n* = 23) compared to singing (*n* = 23) and music listening (*n* = 28) No significant results for other tests	77%
Satoh et al. (2015)	NCT	MMSE RCPM RBMT WF	RCPM: between group improvement in “time to complete” for singing group (*n* = 10) compared to control (*n* = 10) No other significant results	64%
Fraile et al. (2019)	QE	EFCL PSF Cued recall	Cued Recall: pre-post improvement during training period compared to non-training period No significant results for EFCL and PSF (*n* = 12) When outlier was removed (*n* = 11), pre-post improvements in total EFCL and executive processes EFCL during training periods	68%
**Specific word recall**				
Moussard et al. (2014)	QE	Observational data based on: Phase 1: Measured retention of lyrics learnt	Phase 1: Hearing condition had significantly stronger learning effect than shadowing for participants with AD (*n* = 8) Immediate recall: Delayed recall: improved in all sung conditions compared to spoken conditions, with ‘sung, high familiar' conditions being the most effective. Phase 2: Immediate recall: improved across sessions in both spoken and sung conditions	56%
		in spoken vs different type of singing conditions] Phase 2: Measured rate of learning for participants with dementia for spoken and sung non-familiar conditions over 4-week delay	Delayed recall: no overall effect, non-significant trend towards better performance in singing conditions compared to spoken after six sessions. Three participants with AD performed significantly better in sung condition than spoken	
Prickett and Moore (1991)	QE	Video analysis measuring frequency of words recalled and memorised during sung and spoken conditions	Sung lyrics were recalled more frequently than words in spoken conditions Performance was more accurate for singing words to long-familiar songs compared to reciting familiar words, recalling a new song, and reciting a new poem	56%

##### Cognitive Screening Tools

*Seven* studies utilised standardised screening tools to measure cognitive function before and after a singing intervention. Cooke et al. (2010a); Camic et al. (2011); Chen et al. (2019) each used the Mini Mental State Exam (MMSE) and did not report any significant changes overall. Camic et al. (2011) reported no overall significant change, however, they observed MMSE scores varying across participants, with some improving and some deteriorating. Although these results should be interpreted with caution, they do reflect the idiosyncratic nature of dementia progression. Cooke et al. (2010a) and Maguire (2021) found no significant difference within or between groups. Maguire (2021) reported a non-significant trend toward improved MMSE (and a 10-point revised version—R-MMSE) scores for participants in an individualised singing intervention (ISI). They also reported significant improvements for the ISI participants on other cognitive measures (Clock Drawing, Narrative and Complete Sentences), however, these results should be interpreted with caution due to several methodological weaknesses. Chen et al. (2019) found no overall effect of singing on cognition, however, they reported a significant increase on the MMSE recall subscale following a group opera singing intervention.

Wang et al. (2018) used both the MMSE and Montreal Cognitive Assessment (MoCA), and observed significant within group improvements for participants allocated to both singing and standard care, with significantly larger improvements in the singing group. Takahashi and Matsushita (2006) measured cognitive function using the Revised Hasegawa Dementia Scale, and reported that scores for participants receiving a group singing intervention remained stable over a 2-year period, while those in a control group experienced a non-significant decrease. Sub-analysis reported that participants who had initially moderate-high cognitive function at baseline improved their function over the course of the program. However, these results should be interpreted with caution due to the small sample size and non-randomised design. Davidson and Fedele (2011) used the Hierarchical Dementia Scale in a smaller-scale study of group singing, but found no significant changes in cognition.

##### Neuropsychological Batteries

Five studies used a combination of tests to conduct a neuropsychological battery assessment, assessing a range of cognitive abilities. Three RCTs used full neuropsychological batteries (Särkämö et al., 2014; Pongan et al., 2017; Lyu et al., 2018). Särkämö et al. (2014) found that both singing and music listening interventions significantly improved general cognition and attention/executive function in the short term compared to a standard-care control, but only “orientation” remained significantly improved after a 3-month follow-up. Singing was found to have a significant effect on short term/working memory only immediately post-intervention. The authors reported a significant long-term improvement (9 months) in autobiographical recall (i.e., names of people from childhood) in both music conditions, with trends favouring the singing condition.

Similarly, Lyu et al. (2018) reported a significant improvement in semantic verbal fluency immediately following both singing and lyric reading interventions compared to controls, with only the singing group remaining significantly higher at 3 months. They also conducted a sub-group analysis and found that participants with mild stage Alzheimer's demonstrated significantly improved immediate and delayed recall at the conclusion of the singing intervention only, but these improvements were not maintained at 3-month follow up. This may indicate a need for continuous intervention for maintenance of cognitive benefits.

Pongan et al. (2017) found that while verbal memory remained stable for participants in the singing group, decline was observed in a painting control group. Scores on the Digit Span (short-term memory) and Stroop test (processing speed and inhibition) significantly improved for both groups, the latter including a non-significant, but clinically important greater improvement for the singing group. The authors noted that the assessments were completed days or even a week following the final intervention session, which may indicate that these benefits may be longer lasting, and not just occurring due to spontaneous arousal. However, they also concluded that the delay in assessment may also have resulted in non-significant scores on other measures, as the immediate effect of the interventions was not captured.

Two smaller scale studies also used multiple measures for cognition. In a moderate-quality NCT in which participants acted as their own control, Fraile et al. (2019) used the Evaluation Instantane e du Bien-Etre (EFCL) battery, and found that participants who received a 1:1 singing-training program (*n* = 12) improved in the “cued recall” domain only. However, when an outlier was removed (*n* = 11), the authors reported significant improvement in “cued recall” total scores, and in scores on the “executive processes” subscale of the EFCL. Satoh et al. (2015) reported that after 6 months of group singing and home karaoke practise, the only significant change in cognition was improved psychomotor speed (based on scores from the Japanese Raven's Coloured Progressive Matrices measure). A reduction in brain regions required to complete the singing tasks was revealed in Functional magnetic resonance imaging (fMRI) scans, indicating that less cognitive effort was used once participants mastered the signing activity. Although the results in both studies indicate some observable improvement to cognition, it should again be noted that both had small sample sizes and no control, therefore findings should be interpreted with caution.

##### Specific Word-Recall

Two studies examined the effect of a singing-training intervention on participants' ability to recall and memorise new words, using an author-designed intervention and measurement. Moussard et al. (2014) compared the impact of a spoken learning task with singing non-familiar, semi-familiar and high-familiar tunes on learning new words for both participants with Alzheimer's disease and adults with no diagnosis (non-randomised). They found that sung conditions did not influence immediate word recall, but appeared to increase delayed word recall for both participants with Alzheimer's disease and those without. Singing was also observed as slightly advantageous compared to spoken learning conditions after a 4-week period. The authors speculated that this may have been due to singing being more demanding in the initial learning stage leading to improved long-term retention. Prickett and Moore (1991) similarly compared singing to spoken interventions for recall and memory. They found that overall, participants with Alzheimer's disease (acting as their own controls) were able to recall sung lyrics better than spoken lyrics, and that this was improved with highly familiar songs compared to new tunes. The authors also observed that some participants with Alzheimer's disease were able to learn new songs following extended practise, but not spoken poetry, which was shorter in length and contained less words. Despite the small size and non-standardised measures and procedures used in these two studies, the findings provide important clinical insight into the potential mechanisms and effects that singing can have on memory and learning for people living with Alzheimer's disease.

#### Engagement

Eight studies were grouped under this heading examined Two distinct constructs: engagement in singing as an activity, and impact of singing on social engagement (Table 6).

**Table 6:** Overview of results of engagement outcomes.

**Study**	**Design (sample)**	**Outcome Measure**	**Results**	**Quality (D&B)**
**Engagement in singing**				
Harrison et al. (2010)	RCT	Behavioural Checklist measuring engagement (devised by authors)	Active engagement and passive engagement: between group improvement in singing (*n* = 35) compared to reading (*n* = 21)	77%
Groene et al. (1998)	QE* (*n* = 7)	Video analysis using behavioural checklist measuring engagement (devised by authors)	Significantly more “purposeful” responses in the exercise than in singing	47%
Korb (1997)	QE* (*n* = 9)	ABS Bell and Smith's Behavioural Checklist (adapted form)	Unsolicited feedback: significantly more in reminiscence than singing Solicited feedback: significantly more in rhythm and reminiscence than singing Taps to beat: significantly more during rhythm than singing Affect: Between group improvement for singing and rhythm compared to the “reminiscence”	60%
Hanson et al. (1996)	QE* (*n* = 51)	Time-sampling behavioural checklist measuring engagement (devised by authors)	Significantly more “high responses” during movement than during singing, regardless of cognitive function Significantly more “passivity” occurred during singing than during movement	78%
Clair and Bernstein (1990)	QE* (*n* = 6)	Analysis of video observation—measured duration data for (a) vibrotactile response, the drum held in the lap; (b) non-vibrotactile response, the drum held in front of the subject; and (c) singing.	Vibrotactile responses occurred significantly more than non-vibrotactile responses Only one participant engaged in singing at all, significantly less than vibrotactile and non-vibrotactile responses	56%
**Social engagement**				
Davidson and Fedele (2011)	QE (*n* = 27)	Video analysis of behaviours during sessions	Video analysis data revealed high levels of lucidity, engagement, and relaxed affect. during sessions	41%
Lesta and Petocz (2006)	QE (*n* = 4)	Behavioural Checklist measuring engagement (devised by authors)	Flat mood: pre-post improvement (decrease) during session and continued to decrease immediately after Anxious mood: pre-post improvement (decrease) during session, but rose non-significantly immediately post-session Apparent well-being: pre-post improvement during session Non-social behaviour: pre-post improvement (decrease) on most items in checklist during session (mumbling, touching face/clothes, sitting alone, wandering alone), but some increased slightly during immediate period after session Social behaviours: pre-post improvement (increased) across most items (eye contact, smiling, singing, talking, moving to music) and remained high post-session	69%
Olderog Millard and Smith (1989)	QE (*n* = 10)	Bell and Smiths Behavioural Checklist (adapted form)	Frequency of two physical and social behaviours (walking and sitting with others) was significantly higher in the singing condition than in discussion condition Frequency of verbal/vocal participation was significantly higher in the singing condition Frequency of “walking with others” significantly increased following the singing condition	56%

*RCT, Randomised Control Trial; NCT, Non-Randomised Control Trial; QE, Quasi-Experimental Design; ABS, Affect Balance Score*.

##### Engagement in Singing

Five studies compared how participants with dementia engaged in singing interventions (SI) to other musical and non-musical interventions (Clair and Bernstein, 1990; Hanson et al., 1996; Korb, 1997; Groene et al., 1998; Harrison et al., 2010). Three studies found that participants were less engaged in SI than they were in other activities (including movement, drumming, and discussion group) (Clair and Bernstein, 1990; Korb, 1997; Groene et al., 1998). The different level of cognitive and social demands of each activity were raised as potential reasons for this difference. Korb (1997) suggested that increased verbal feedback in their discussion group was likely due to more opportunities for comments in comparison to the music interventions. Clair and Bernstein (1990) and Groene et al. (1998) each reported that their control interventions (rhythm and movement, respectively) were less cognitively demanding, and may therefore have been easier for participants to engage in. Participants in these studies were reported to be experiencing moderate-severe cognitive challenges as a result of their dementia progression, which may have impacted their ability to engage in verbal aspects of singing. However, the sample sizes for these studies were small, non-standardised measures were used, and quality varied from fair-low (Table 6). Therefore, these results should be interpreted cautiously.

In a moderate-quality NCT (*N* = 51), Hanson et al. (1996) similarly compared movement, rhythm and singing interventions at different levels of intensity, and found that participants were able to engage actively in singing (and rhythmic interventions) at a low-demand level, but were less able when the task became more demanding. They also observed that participants in the singing groups engaged “passively” significantly more than in other groups. The authors found that participants were able to engage in less cognitively demanding activities (particularly movement) at higher intensity and postulated that this may have been due to the differences in the cognitive demands of each task and that some types of activities were beyond the ability of some participants (Hanson et al., 1996). A similar sized (*N* = 47) moderate-quality RCT reported a significant increase in both active and passive engagement in a SI compared to a reading control group (Harrison et al., 2010). Although Harrison et al. reported more positive results for the SI than other included studies, this may be accounted for by the difference in control interventions; a reading group is possibly more cognitively demanding for participants than singing, and may not encourage the same level of interaction compared to drumming or movement activities. Notably, participants in the study by Harrison et al. (2010) were reportedly in the early-mid stages of dementia, with a MMSE score indicating mild-moderate cognitive challenges. In contrast, Hanson et al. (1996) included participants with mild-severe cognitive challenges. Camic et al.'s (2011) pre-post study suggested that even participants with moderate-severe cognitive challenges were able to engage in group singing. However, the authors did not provide details of the nature of the engagement (i.e., active or passive), and participants were still living in the community, whereas Hanson et al. (1996) included participants in a range of settings (from community day centres to residential and Alzheimer's specific wards). It is therefore reasonable to conclude that the stage of dementia and level of cognitive challenge may impact an individual's ability to engage in singing as an activity.

##### Social Engagement

Three studies used observational checklists to measure changes in behaviour that indicated social engagement (Olderog Millard and Smith, 1989; Lesta and Petocz, 2006; Davidson and Fedele, 2011). All three studies reported that participants demonstrated increased social engagement either during or following SI. Olderog Millard and Smith (1989) observed an increase in “walking and sitting with others,” and verbal or vocal engagement during the SI (with “walking with others” remaining high post session). Lesta and Petocz (2006) similarly reported increased social behaviours during and following SI, and decreased non-social behaviours during the SI. An exploratory pre-post study measured within-session behaviour using an observational checklist and found that lucidity, energy, and on-task focus increased during the SI (Davidson and Fedele, 2011). Although the findings from these studies generally suggest SI improves social engagement, the sample sizes were small and reporting quality was low. Additionally, the measures were not always standardised, and the construct of what constitutes social engagement was not always clear.

#### Activities of Daily Living

Five studies measured the impact of singing on Activities of Daily Living (ADL) for people living with dementia (Table 7) (Camic et al., 2011; McHugh et al., 2012; Satoh et al., 2015; Lyu et al., 2018; Hiller, 2020). Three studies used standardised measures to examine the effect on overall ADL. One high quality RCT found no significant change in ADLs following a group singing intervention or reading control (Lyu et al., 2018). Two smaller-sized pre-post studies also found no significant change in ADL scores, however, both observed a non-significant trend toward ADLs decreasing, which authors explained as an expected progression of dementia (Camic et al., 2011; Satoh et al., 2015).

**Table 7:** Overview of results of activities of daily living outcomes.

**Study**	**Sample**	**Outcome measure**	**Results**	**Quality (D&B)**
Lyu et al. (2018)	RCT	Barthel Index	No significant change (*n* = 288)	85%
McHugh et al. (2012)	RCT	1. Data relating to percentage of food intake recorded by staff 2. Video observation during meal time (to supplement data recorded by staff)	No significant change (*n* = 15)	81%
Satoh et al. (2015)	NCT	Barthel index IADL	No significant change (*n* = 20)	64%
Hiller (2020)	QE	Plates of food were weighed pre-post to measure food intake	No significant change (*n* = 28)	73%
Camic et al. (2011)	QE	BADLS	No significant change (*n* = 10)	70%

*RCT, Randomised Control Trial; NCT, Non-Randomised Control Trial; QE, Quasi-Experimental Design; BADLS, Bristol Activities of Daily Living Scale; IADL, Instrumental Activities of Daily Living Scale*.

Two studies measured the effect of group singing immediately prior to mealtime on the food or nutritional intake of participants with dementia who lived in aged-care. An RCT (*n* = 15) found no significant change, and attributed this to small participant numbers and inconsistencies in study adherence (McHugh et al., 2012). A pre-post study (*n* = 28) similarly noted no significant change, however, they also observed that food intake was greater during the baseline measurements than following intervention for residents at two out of three facilities (Hiller, 2020). The authors speculated that this could potentially be related to an increase in serotonin (due to singing), which has been known to suppress appetite, although owing to small sample sizes, this warrants further investigation.

#### Care-Partner Outcomes

Five studies included measures to specifically investigate the impact of singing for family care-partners (Table 8). Studies by Camic et al. (2011); Särkämö et al. (2014); Mittelman and Papayannopoulou (2018), and Tamplin et al. (2018), included group singing for participants with dementia and care-partner dyads. Alternatively, Satoh et al. (2015) interviewed care-partners who did not participate in the intervention themselves. A range of outcome measures focused on general health, mental health, quality of life, self-perception, relationship between care-giver and care-recipient, and aspects of caregiving (such as perceived “burden,” and positive aspects) (Table 8). Only two studies reported a significant improvement for care-partners. Särkämö et al. (2014) reported a significant decrease in perceived care-partner burden following participation in group singing with their care-recipient. Mittelman and Papayannopoulou (2018) reported a significant increase in care-partner self-esteem, and a trend toward increased social support. They also reported that despite not observing changes for depression, baseline scores were high, suggesting a ceiling effect. Satoh et al. (2015) did not observe any change in perceived burden scores, but recognised this lack of deterioration as important alongside care-partner reports of decline in the ability of their partners to complete activities of daily living (ADLs), which could conceivably affect their perceived burden. Similarly, Camic et al. (2011) reported ceiling effects for care-partners in relation to QOL and mood (stress, anxiety and depression). It is difficult to draw conclusions about this category due to the heterogeneity of the outcomes that were measured. However, the positive result from a moderate-quality RCT (*n* = 84) (Särkämö et al., 2014), and lack of deterioration in other studies suggest that further investigation into the potential benefits for care-partners is warranted.

**Table 8:** Overview of results for care-partner outcomes.

**Study**	**Design**	**Outcome measure**	**Results**	**Quality (D&B)**
Särkämö et al. (2014)	RCT	ZBI GHQ	ZBI: between group improvement (decrease) for singing (*n* = 27), compared to music listening (*n* = 29) and control (*n* = 28) at long-term follow up (9 months) GHQ: no significant change	77%
Satoh et al. (2015)	NCT	ZBI	No significant change (*n* = 20)	64%
Tamplin et al. (2018)	QE	QPCR PHQ9 SWLS PACQ FS	No significant changes, possible floor/ceiling effects on QPCR and FS (*n* = 9)	78%
Mittelman and Papayannopoulou (2018)	QE	MOS-SSS SF-8 GDS RSS C_FAM	RSS: pre-post improvement (*n* = 11) No significant changes for other outcomes *significance set at *P* < 0.1	69%
Camic et al. (2011)	QE	DASS WHO-QoL BREF	No significant change, possible floor/ceiling effect (*n* = 10)	70%

*RCT, Randomised Control Trial; NCT, Non-randomised Control Trial; QE, Quasi-Experimental Design; DASS, Depression, Anxiety, Stress Scales; WHO-QoL BREF, Abbreviated World Health Organisation Quality of Life Questionnaire; MOS, Medical Outcomes Study; SF-8, Medical Outcomes Survey Short Form-8 Questionnaire; GDS, Geriatric Depression Scale; GHQ, General Health Questionnaire; ZBI, Zarit Burden Interview; QPCR, Quality Carer-Patient Relationship scale; PHQ-9, Patient Health Questionnaire 9; SWLS, Satisfaction with Life Scale; PACQ, Positive Aspects of Caregiving Questionnaire*.

#### Conclusion for Synthesis of Quantitative Data

Across the seven categories identified in this narrative synthesis, heterogeneity of outcomes, settings, participant demographics, and quality of studies made it difficult to draw concrete conclusions about the impact that singing can have for people with dementia and their care-partners. However, the positive results in each category suggest that effects may be present, but difficult to capture, particularly where baseline scores indicated good health or well-being, or lack of change indicated no decline in a time where decline would be expected. Further research into these outcomes is warranted, however, methodological challenges may need addressing in order to capture the impact of complex phenomenon.

### Thematic Synthesis of Qualitative Findings

Twelve studies included qualitative data regarding the experience of singing for people living with dementia (and in some cases, their care-partners). Two studies reported using the same data (Clark et al., 2018; Tamplin et al., 2018); therefore, these data sets were considered to be one study. The study designs and methods varied considerably: Table 9 depicts the method and design of each included study. All of the studies featuring qualitative data included group singing interventions. The thematic synthesis produced seven key themes that were represented across the included studies (Table 10).

**Table 9:** Summary of qualitative study design.

**Study**	**Study design**
Camic et al. (2011)	Thematic analysis of interview data (individual interviews)
Clark et al. (2018)	Thematic analysis of dyad interview data
Dassa and Amir (2014)	Content analysis of session transcripts
Davidson and Almeida (2014)	Brief qualitative interviews, presented as quotes in table with corresponding quantitative data
Davidson and Fedele (2011)	Anecdotal feedback collected and documented in writing throughout program, presented without analysis alongside corresponding quantitative data
Hara (2011)	Ethnographic research
Harris and Caporella (2014, 2018)	Thematic analysis of interview data (focus groups)
Lee S. et al. (2020)	Interpretative phenomenological analysis (IPA) of interview data
McCabe et al. (2015)	Thematic analysis of interview data (individual, dyad or small group interviews)
Mittelman and Papayannopoulou (2018)	Notes written during focus group interviews, presented without analysis
Osman et al. (2016)	Thematic analysis of interview data (individual/dyad interviews)
Tamplin et al. (2018)	Thematic analysis of interview data (dyad interviews)
Unadkat et al. (2016)	Grounded Theory analysis of interview data

**Table 10:** Examples for qualitative themes and subthemes.

**Theme/Subtheme**	**Example of qualitative data**
**Theme 1: Pragmatic elements of the sessions shaped the experience**	
*Subtheme 1.1: Singing Is Accessible*	“…even if you can't sing. Other people do that and even if they're very old or young they can still do something like that.” (participant with dementia, quoted in Unadkat et al., 2016). “I think it brings a lot of people to a same level, you know, so that everyone's the same. We're all singing.” (care-partner, quoted in Unadkat et al., 2016) “I see people there that are very, very ‘far gone', and yet I see them participating, which I think is wonderful” (care-partner, quoted in Osman et al., 2016)
*Subtheme 1.2: Intentional design elements made programs accessible*	“…they have structured the whole thing around the needs of these people, very much with them as the centre and the focus.” (care-partner, quoted in Unadkat et al., 2016)
*Subtheme 1.3: Role of the Facilitator*	“…they went to [local dementia organisation] for advice on how to deal with it… because the ones that were toiling with it were the musicians, but they had to get advice from organisations that's used to dealing with people with behavioural issues, and it got sorted' (care-partner, quoted in McCabe et al., 2015)
*Subtheme 1.4: Sustainability*	“Everybody's talking about when's it going to finish. And they're not just talking about it—they're really concerned about it. I know it's research—and I know it's incredibly important and I think it's wonderful that it's happening, but I think it's such a shame that when the people are in the here and now, that they're actually benefitting from it. It's like being given a trial drug and then it fixes you but you can't keep going” (care-partner, quoted in Clark et al., 2018)
*Subtheme 1.5: Getting Involved*	“We were very apprehensive” “I don't sing and if it hadn't been for my husband, I would not have dreamed of going” (care-partners, quoted in Camic et al., 2011)
**Theme 2: Social benefits of group singing**	
*Subtheme 2.1: Group singing fosters a sense of connexion and belonging*	“…singing seems to break down barriers and to open up sort of, not only companionship, but a sense of belonging, and that's great” (participant with dementia, quoted in Osman et al., 2016)
*Subtheme 2.2: Social Support*	“…because you know that the person over there has the same sort of problems I have, and the person sitting beside you, you can talk about it” (participant with dementia, quoted in Lee S. et al., 2020)
*Subtheme 2.3: Increased Social Engagement*	“She enjoyed doing something normal with other people. We both did. She has become more engaged with other activities” (care-partner, quoted in Camic et al., 2011)
**Theme 3: Singing impacts mood**	
*Subtheme 3.1: Singing is enjoyable in the moment*	“As time passes and you get to know people more, you can see the singing shaping their mood. Someone comes in grumpy and leaves happy and smiling. I work to achieve those sort of outcomes” (facilitator, quoted in Davidson and Almeida, 2014)
*Subtheme 3.2: Singing improves Mood Explicitly*	“We go away feeling uplifted, the lightness and brightness follows us home…” (care-partner, quoted in Unadkat et al., 2016)
**Theme 4: Participating in singing groups impacts sense of identity**	
*Subtheme 4.2: Sense of Fulfilment*	“…it made you feel that you were important, which is important in itself” (participant with dementia, quoted in Unadkat et al., 2016)
*Subtheme 4.3: Connecting to Other Parts of Identity*	“It's something to live for. I was still a little bit less than I am now – in being able to find the words and things—and the first day we went, [another participant] they were anxious. You could tell, and somehow or other I was just able to talk to one of them. I was really thrilled, because that was me” (participant with dementia, quoted in Clark et al., 2018)
**Theme 5: Benefits to memory**	“…they [the songs] were all coming back to me [during the sessions]” (participant with dementia, quoted in Lee S. et al., 2020) “Aye, usually at our state right now you forget things quite quickly. We've never forgot the theatre group which is strange. Because I remember still the same…I can feel it.” (participant with dementia, quoted in McCabe et al., 2015)
**Theme 6: Flow on effects of community group singing**	
*Subtheme 6.1: Change in Routine*	“She enjoyed doing something normal with other people. We both did. She has become more engaged with other activities” (care-partner, quoted in Camic et al., 2011)
*Subtheme 6.2: Building Resources*	“So, I learned out of it as well, about how to get my dad involved in things, where we're both involved, and get him to get the most out of it…” (care-partner, McCabe et al., 2015)
*Subtheme 6.3: Ripple Effects*	“Some people exchanged e-mail addresses, and some of the couples made plans to meet for lunch a few weeks later” (author observations in Harris and Caporella, 2014)
**Theme 7: Singing together supports care-partner relationships**	
*Subtheme 7.1 Opportunity for change in relationship dynamics*	“it was some- thing we could do together, where I wasn't responsible…I was relieved of any responsibility as it was all taken care of.” (care-partner, quoted in Unadkat et al., 2016)
*Subtheme 7.2 Shared experiences help to maintain relationship*	“was excited when I learnt that I could also be involved… It wasn't just – did my mum want to – but do you both want to… it was something that we could do together that was a happy thing” (care-partner, Clark et al., 2018)
*Subtheme 7.3 Care-partners benefit from seeing partner benefit*	“The thing I've noticed about this experience is how it shows him in a good light. I often look at him and think “that's not the man I married.” But then since I've been hearing him singing and looking and behaving like a normal man and I think “that's him, he's still my husband in there.” (care-partner, quoted in Davidson and Almeida, 2014)

#### Theme 1: Pragmatic Elements of the Sessions Shaped the Experience

Eleven studies featured responses that related to how the pragmatic elements of the various singing groups or choirs shaped the experience for participants (Camic et al., 2011; Davidson and Fedele, 2011; Hara, 2011; Davidson and Almeida, 2014; McCabe et al., 2015; Osman et al., 2016; Unadkat et al., 2016; Clark et al., 2018; Mittelman and Papayannopoulou, 2018; Tamplin et al., 2018; Lee S. et al., 2020).

##### Subtheme 1.1: Singing Is Accessible

Singing was perceived as something that participants could do regardless of their diagnosis or past musical experience. Participants with dementia and care-partners could both participate in singing, thereby creating a sense of equality between them (Camic et al., 2011; Hara, 2011; McCabe et al., 2015; Unadkat et al., 2016; Lee S. et al., 2020). Group singing afforded a sense of safety because participants could blend in and not stand out (Camic et al., 2011; Hara, 2011; Unadkat et al., 2016; Clark et al., 2018; Mittelman and Papayannopoulou, 2018), and autonomy to choose their degree and type of participation (McCabe et al., 2015; Lee S. et al., 2020). Davidson and Fedele (2011) noted that some participants needed support to join in and while others were able to participate independently.

##### Subtheme 1.2: Intentional Design Elements Made Programs Accessible

Included studies described how the design elements of each program fostered accessibility (or not). Practical/logistical elements made the programs accessible. For example, group size, length/timing, location/venue, repertoire, and materials used with sessions etc. (Camic et al., 2011; Davidson and Fedele, 2011; Hara, 2011; McCabe et al., 2015; Osman et al., 2016; Unadkat et al., 2016; Clark et al., 2018; Lee S. et al., 2020). Participants observed staff or volunteers going out of their way to ensure that the program was welcoming (Hara, 2011). This, plus the fact that members shared similar experiences was also seen to create a sense of safety or security within the group (Camic et al., 2011; Hara, 2011).

##### Subtheme 1.3: Role of the Facilitator

The facilitator's approach encouraged active participation (Unadkat et al., 2016), created a safe space (Camic et al., 2011; Hara, 2011; Clark et al., 2018) and generally brought a positive energy to the group. Unadkat et al. (2016) highlighted that the facilitator is key to allowing the benefits described in other themes to occur. Participants described how facilitators who were not trained to work with people with dementia experienced challenges in making singing accessible and that training led to better support for people with dementia (McCabe et al., 2015).

##### Subtheme 1.4: Sustainability

Five studies included in this review included singing groups established for research (Harris and Caporella, 2014, 2018; McCabe et al., 2015; Clark et al., 2018; Mittelman and Papayannopoulou, 2018). The importance of program sustainability post-research was noted (McCabe et al., 2015; Clark et al., 2018; Mittelman and Papayannopoulou, 2018). Some participants expressed concern about the future of the singing group (Clark et al., 2018), the negative impact concluding the group had on some participants, and the importance of considering closure and sustainability for future projects (McCabe et al., 2015). Mittelman and Papayannopoulou (2018) initially intended for the program to be short term, however, due to the positive response from participants, it was continued post-study.

##### Subtheme 1.5: Getting Involved

Two studies contained themes relating to the experience of registering for the programs. Participants described a range of feelings relating to joining, from enthusiasm due to love of music, to hesitance due to inexperience or perceived lack of musical ability (Camic et al., 2011). McCabe et al. (2015) described some barriers participants faced (e.g., lack of access to information about the program), and observed that musical preference motivated some participants to join.

#### Theme 2: Social Benefits of Group Singing

Ten of the 11 studies contained themes or participant comments relating to social benefits of group singing for people living with dementia and care-partners.

##### Subtheme 2.1: Group Singing Fosters a Sense of Connexion and Belonging

Participants in the group singing programs experienced a sense of connexion and belonging with other group members (Camic et al., 2011; Hara, 2011; Dassa and Amir, 2014; Harris and Caporella, 2014, 2018; Osman et al., 2016; Unadkat et al., 2016; Clark et al., 2018; Mittelman and Papayannopoulou, 2018; Lee S. et al., 2020). Participants suggested that singing enabled participants to connect with one another through the sharing of experiences of dementia (Camic et al., 2011; Clark et al., 2018; Lee S. et al., 2020). It was suggested that the act of singing itself fostered these connexions and enabled participants with varying abilities and experiences to connect (Hara, 2011; Unadkat et al., 2016; Mittelman and Papayannopoulou, 2018). Performing together similarly strengthened bonds (McCabe et al., 2015). One participant reported that the sense of connexion experienced in the singing groups was deeper than that which they had experienced in a typical support group setting (Osman et al., 2016).

##### Subtheme 2.2: Social Support

Six studies (Camic et al., 2011; Hara, 2011; Osman et al., 2016; Clark et al., 2018; Mittelman and Papayannopoulou, 2018; Lee S. et al., 2020) reflected the specific benefit for care-partners: experiencing social support from attending the singing groups with their loved one who had a diagnosis of dementia. This support appeared to have two key effects/features:

a) Empathy and understanding (Camic et al., 2011; Hara, 2011; Osman et al., 2016; Clark et al., 2018; Lee S. et al., 2020): Care-partners felt a sense of comfort knowing they were able to seek support from others experiencing similar situations. One participant commented that they appreciated having a shared understanding without specifically having to talk about diagnoses or illness (Camic et al., 2011).
b) Knowledge and Resources (Hara, 2011; Osman et al., 2016; Mittelman and Papayannopoulou, 2018): Some participants spoke of being able to share and receive information about what to expect in the progression of dementia, and resources that other people had found useful.

##### Subtheme 2.3: Increased Social Engagement

Reflections were offered about how the singing groups enabled engagement in a social activity when other social activities were inaccessible (Camic et al., 2011; Hara, 2011; Harris and Caporella, 2014; Osman et al., 2016). Participants in one study described enjoying the opportunity to meet people of different ages in their intergenerational choir, as they “usually don't have a chance to be with so many young people” (Harris and Caporella, 2014, p. 278). Others described a sense of invigoration when engaging with others, with one participant describing how their partner who had dementia would “come to life when [they were] in company” (Camic et al., 2011, p. 169).

#### Theme 3: Singing Impacts Mood

The ways that group singing impacted participants' mood was present across all studies.

##### Subtheme 3.1: Singing Is Enjoyable in the Moment

Singing was perceived as an enjoyable activity by participants (Camic et al., 2011; Davidson and Fedele, 2011; Davidson and Almeida, 2014; Harris and Caporella, 2014; McCabe et al., 2015; Osman et al., 2016; Unadkat et al., 2016; Clark et al., 2018; Mittelman and Papayannopoulou, 2018; Lee S. et al., 2020). Three studies featured a specific theme of singing being enjoyable (Camic et al., 2011; Unadkat et al., 2016; Clark et al., 2018), while the notion of enjoyment was frequently represented in quotes from participants or reflections by authors in other studies. Unadkat et al. (2016) theorised that enjoyment may relate to “in-the-moment” experience of pleasure, or transient improvement to mood. This is consistent with other studies, who reported that participants experienced a state of flow during group singing (Hara, 2011; Clark et al., 2018).

##### Subtheme 3.2: Singing Improves Mood Explicitly

Nine studies explicitly reported that singing improved mood (Camic et al., 2011; Hara, 2011; Harris and Caporella, 2014, 2018; Osman et al., 2016; Unadkat et al., 2016; Clark et al., 2018; Mittelman and Papayannopoulou, 2018). Participants experienced or observed an improvement in mood beyond the session (Camic et al., 2011; Hara, 2011; Osman et al., 2016; Unadkat et al., 2016; Ward and Parkes, 2017; Clark et al., 2018; Mittelman and Papayannopoulou, 2018) although the length of this improvement was not always described (Davidson and Fedele, 2011; Dassa and Amir, 2014). Singing was recognised by some as a preferable option to pharmacological treatment to address mood (Unadkat et al., 2016; Mittelman and Papayannopoulou, 2018).

#### Theme 4: Participating in Singing Groups Impacts Sense of Identity

Several studies reported ways that group singing positively impacted participants' sense of identity.

##### Subtheme 4.1: Increased Confidence for Participants With Dementia

Studies reported an increase in confidence for participants with dementia as a result of the group singing programs (Camic et al., 2011; Hara, 2011; Dassa and Amir, 2014; McCabe et al., 2015; Clark et al., 2018; Mittelman and Papayannopoulou, 2018). One participant reported that the singing groups facilitated their coming to terms with their diagnosis (Osman et al., 2016). This is notable as the presence and impact of stigma relating to a dementia diagnosis is common (Harris and Caporella, 2014, 2018). Some participants with dementia reported that group singing challenged their own negative self-perception (Camic et al., 2011; Davidson and Fedele, 2011; Clark et al., 2018), which helped them to realise they ‘can still do a lot of things' (McCabe et al., 2015).

##### Subtheme 4.2: Sense of Fulfilment

Participants experienced a sense of achievement or fulfilment from being involved in the singing groups (Camic et al., 2011; Davidson and Fedele, 2011; Dassa and Amir, 2014; Unadkat et al., 2016; Clark et al., 2018; Lee S. et al., 2020). They felt that they were contributing to “something worthwhile” (Camic et al., 2011, p. 168), which made them feel “important” and “valued” (Unadkat et al., 2016, p. 475). Participants reported enjoying being able to contribute to music therapy students' education by helping them on their placement, feeling that they were contributing to something bigger than themselves (Clark et al., 2018). One participant reported feeling proud of being in the group (Clark et al., 2018), while others demonstrated pride by inviting others to witness it (Davidson and Fedele, 2011). The creation of a finished product was also valued as an achievement (Unadkat et al., 2016; Lee S. et al., 2020).

##### Subtheme 4.3: Connecting to Other Parts of Identity

Studies reported that group singing programs afforded opportunities for people with dementia to connect to their past identities (Camic et al., 2011; Clark et al., 2018). Singing provided a sense of “normalcy” and connexion to a pre-illness identity for participants with dementia and care-partners, with one care-partner describing this as a return to their “old self” (Camic et al., 2011; Mittelman and Papayannopoulou, 2018). Other studies reported that the groups enabled people with dementia to showcase their musical skills and consequently be seen by others in a different light (Davidson and Almeida, 2014; Lee S. et al., 2020).

Participants who had a pre-existing relationship with singing or music reported a re-connexion with their musical identity during the group singing programs (Hara, 2011; Unadkat et al., 2016; Clark et al., 2018). Some participants also described a re-framing or shift in their identity, including musician as a new identity, or as an alternative to a person with a diagnosis (Hara, 2011). One care-partner described enjoying the opportunity to connect with their own musicality during the program (McCabe et al., 2015). Participants' musical engagement outside of the groups increased following participation, which may indicate they were adopting a growing musical identity outside of the group (Camic et al., 2011; Mittelman and Papayannopoulou, 2018).

#### Theme 5: Benefits to Memory

Participants with dementia and care-partners reported some improvements to memory while participating in group singing programs. Some care-partners and facilitators reported observing members with dementia being able recall lyrics from memory, learn new songs (Davidson and Fedele, 2011; Osman et al., 2016; Clark et al., 2018), or recall the program week to week or after it had concluded (Davidson and Fedele, 2011; McCabe et al., 2015; Clark et al., 2018). Some participants with dementia expressed surprise at their ability to do this, given their ongoing challenge with recall (McCabe et al., 2015). Music-stimulated reminiscence (Davidson and Fedele, 2011; Dassa and Amir, 2014; Clark et al., 2018) and autobiographical recall of memories were often reported. Dassa and Amir (2014) also found that some songs prompted memory and reflection of world or cultural events.

#### Theme 6: Flow on Effects of Community Group Singing

Several studies included themes relating to flow on effects of community group singing programs for people living with dementia and their care-partners.

##### Subtheme 6.1: Change in Routine

Regular attendance at the singing groups helped participants to develop more structure in their week (Camic et al., 2011; Hara, 2011; Mittelman and Papayannopoulou, 2018). For example, attending a choir helped participants discover new activities or routines to do in relation to the program (e.g., feeding swans on way to choir) (Hara, 2011).

##### Subtheme 6.2: Building Resources

Relationships enabled by group participation were important for participants to build resources. Some care-partners indicated that being involved in the groups enhanced their capacity to provide care through improved personal well-being (Osman et al., 2016), while others learned new caregiving skills through interaction with other group members (McCabe et al., 2015). Community-based groups played an important role in providing resources and information about other available supports (Hara, 2011; Unadkat et al., 2016).

##### Subtheme 6.3: Ripple Effects

Some studies reported that there were ecological benefits derived from connexions formed during sessions that extended beyond the groups; this included new friendships, socializing, and supporting each other outside of the programs (Hara, 2011; Clark et al., 2018). Some participants felt that performing (McCabe et al., 2015) or participating in intergenerational choirs (Harris and Caporella, 2014, 2018) played an important role in advocacy—educating relatives/friends and the wider public about dementia.

#### Theme 7: Singing Together Supports Care-Partner Relationships

Several studies described ways that singing together benefits care-partners as well as people with dementia.

##### Subtheme 7.1 Opportunity for Change in Relationship Dynamics

Singing groups provided opportunities for care-partners and participants with dementia to experience changes in their relationship “role.” For care-partners, the groups provided a chance to be temporarily released from their caring responsibilities, and to participate as an equal with their partner (Hara, 2011; Unadkat et al., 2016). In such situations, participants with dementia experienced opportunities to be the expert in the relationship, particularly when they were more musically experienced than their care-partner (Unadkat et al., 2016).

##### Subtheme 7.2 Shared Experiences Help to Maintain Relationship

The shared experience of singing together as a dyad provided participants with a way to connect meaningfully and maintain aspects of their relationship that could be challenged by the progression of dementia. One participant described the importance of having a meaningful activity that her father was able to engage in McCabe et al. (2015). Singing groups provided dyads who had previously participated in music together an accessible way to continue this aspect of their relationship (Clark et al., 2018). Conversely, participating together allowed one member of the dyad, the chance to experience the other members' interests. For example, some participants with dementia were able to share in their care-partner's love of music for the first time in their relationship (Hara, 2011; Clark et al., 2018). Attending together gave participants a shared interest to talk about (Lee S. et al., 2020). Further, within the music itself, participants were able to connect and acknowledge the shared experience in the moment without the need to verbalise what they were experiencing (Harris and Caporella, 2014; Osman et al., 2016).

##### Subtheme 7.3 Care-Partners Benefit From Seeing Partner Benefit

Some care-partners described that co-participation in the singing groups provided them with space to witness their family member living with dementia flourish. For some, this was experienced as perceiving their loved one acting like their “old self” (Hara, 2011; Unadkat et al., 2016). This benefit was motivating enough for care-partners who did not particularly enjoy the singing themselves to attend (Camic et al., 2011; Hara, 2011).

#### Conclusion for Synthesis of Qualitative Data

The thematic synthesis highlighted several perceived benefits of group singing experienced by participants with dementia and their care-partners. The quality of the included studies was variable, with several studies not including their analysis methods in explicit enough detail to assess the trustworthiness of the reporting. At times, data was not analysed at all, and reports were based on informal comments from participants rather than formalised interviews. The perspectives of caregivers, familial and professional, tended to dominate the data, even though participants with dementia were included in several studies. Further studies would benefit from a more structured approach to data collection to ensure that equal weight is given to the perspectives of people with dementia and their care-partners.

### Meta-Integration—Mixed Studies Synthesis

Following the independent synthesis of qualitative and quantitative results, the first author conducted a third process, synthesising the themes that emerged from the initial syntheses together to compare, contrast and integrate the findings to give a fuller picture. Four key areas were identified from this synthesis: psychological well-being, quality of life, cognition, and care-partner experience.

#### Psychological Well-Being

The clearest link between the quantitative outcomes and themes reported in the qualitative studies was the impact of group singing on psychological well-being. Several quantitative studies measured mood-related disorders, while the qualitative synthesis revealed that participants experienced in-the-moment and delayed benefits to mood. Despite the prevalence of benefits to mood captured in the thematic synthesis, the quantitative data synthesis indicated mixed results in relation to the potential for singing to improve psychological well-being. The methodological quality, heterogeneity of outcome measures, and floor and ceiling effects may have contributed to the lack of statistically significant results in this category. Notably, the qualitative studies did not report participants describing their psychological states in a particularly pathological way. Alternatively, the benefits to mood were described in a more positive light; for example, participants used words such as “enjoyable,” “uplifting,” and being able to “switch off.” It is possible that participants did not have the language or inclination to discuss their psychological well-being using recognised medical terminology. However, it is also possible that while they did not identify as experiencing extreme psychological anguish, the positive experiences of the group singing still provide an uplifting boost to their psychological well-being. This was pertinent in the two papers reporting on the Remini-Sing study (Clark et al., 2018; Tamplin et al., 2018). In their reporting of quantitative data, they found no significant improvement in depression and agitation, but also noted floor/ceiling effects. However, their qualitative results reported themes relating to enjoyment and positive personal well-being. This might indicate that group singing could have a positive impact on mood but may be difficult to measure if participants do not have a notable level of psychological distress. There is a growing understanding in the positive psychology literature that the absence of psychological distress does not equate to positive well-being (Huppert, 2009). In several studies, authors speculated that the lack of deterioration observed in mood-related outcomes may signify that the singing groups acted as a buffer or prophylactic against increasing depression or anxiety, as could typically be expected with dementia disease progression (Cooke et al., 2010b; Camic et al., 2011; Tamplin et al., 2018). Regular sessions may be required to maintain positive effects to mood or psychological well-being, as no long-term benefits were observed at three-month follow-up (Särkämö et al., 2014, 2016).

These findings demonstrate an interaction between the experience of group singing and perception of mood, and suggest that transitory benefits may be experienced, and a longer-term preventative effect may also be at play where regular sessions provide a form maintenance for psychological well-being. This reflects previous work suggesting that engagement in meaningful activities and social opportunities can enhance the well-being of a person with dementia (Kitwood, 1997; Snyder, 2006) and prevent psychological deterioration (Santos et al., 2013). However, given the methodological challenges in the quantitative studies, and non-generalisable nature of the qualitative studies, further research into this phenomenon is required to fully understand how this interaction can support people who may be experiencing significant psychological distress.

#### Quality of Life

Quality of life, as a distinct concept, was not an explicit feature of the qualitative results. However, several themes or subthemes from the thematic synthesis related to different aspects of QOL. Each of the main measures used in the quantitative studies (DemQOL, QOL-AD, and EQ-5D) measure QOL across constructs relating to physical and psychological health, relationships, independence, and cognition. Factors including mood/emotional state, cognitive ability, independence to complete activities of daily living, and communication ability have also been noted to impact QOL for people with dementia (Kwasky et al., 2010). However, other indicators of QOL for general populations include interpersonal relationships, social inclusion, personal development, and self-determination (World Health Organization, 1998). Reduced opportunities for meaningful connexion, and subsequent diminished sense of personhood, have also been described as having potential to exacerbate the negative life experiences for people with dementia (Kitwood, 1997; Snyder, 2006).

With these understandings of QOL in mind, the qualitative themes (and subthemes) of social connexion, support and engagement, identity, and flow on effects indicate the ways that group singing has contributed to domains of QOL for participants with dementia and their care-partners. These qualitative findings provide some important context in light of the quantitative results. Although only three studies reported significant improvement in QOL (Pongan et al., 2017; Cho, 2018; Mittelman and Papayannopoulou, 2018), others reported either significant improvement on individual scale items, or no deterioration of QOL following initial high scores. For example, some studies reported increase in items relating to friendship, mood, enjoyment (Chen et al., 2019) and living situation (Davidson and Fedele, 2011). These individual items reflect some of the benefits that participants have described in the qualitative themes.

The thematic analysis also revealed how some people found the group impacted their sense of identity in relation to a sense of confidence, fulfilment, and connecting to past identity. This qualitative finding may help to explain results from Cooke et al. (2010a), who observed an increase on the “self-esteem” QOL item in both singing and reading control groups. Being engaged in meaningful activities has been reported to be an important factor in supporting people living with dementia to maintain their sense of self (Kitwood, 1997; Snyder, 2006). It is possible that in the Cooke et al. (2010a) study, both the reading control group and the singing group fulfilled an unmet need of participants in relation to being engaged in a meaningful activity. Some participants in the qualitative studies described a sense of pride in their membership of the group, due to the teamwork that was inherent in the group-singing activity (Unadkat et al., 2016). Notably, some programs that featured performance opportunities described an additional sense of achievement (McCabe et al., 2015; Unadkat et al., 2016), however this was not frequently reported in these papers. Given that a sense of purpose and achievement have been identified as important factors in maintaining self-hood (Snyder, 2006), future research could focus on the potential benefits of performance.

As with the quantitative outcomes for psychological well-being, several studies measuring QOL reported initial high baseline scores that did not deteriorate over time (Cooke et al., 2010a; Camic et al., 2011; Tamplin et al., 2018). Again, this may indicate the potential for singing groups to act as a protective measure in supporting QOL. Qualitative themes support this hypothesis, as participants described the positive impact that group singing had on various domains related to QOL. Only one study measured the impact group singing programs on care-partners (Camic et al., 2011). Further research can also investigate the potential for care-partners to experience benefits to QOL while participating together with their loved one.

#### Cognition

Cognition, or related aspects such as memory, featured prominently in both the quantitative and qualitative synthesis. The quantitative synthesis revealed mixed results, with only one medium quality study showing improvements when using a cognitive screening tool (Wang et al., 2018). Other studies using a full neuropsychological battery found improvements on specific aspects of cognition (Särkämö et al., 2014; Satoh et al., 2015; Pongan et al., 2017; Lyu et al., 2018; Fraile et al., 2019), and two small-scale studies found some aspects of learning improved in 1:1 singing conditions (Prickett and Moore, 1991; Moussard et al., 2014). In the thematic synthesis, themes relating to cognition described improved memory related to learning (both of the songs/lyrics, and of the routine of attending the program) and reminiscence. Changes to cognition were also alluded to in the way that participants described increased confidence in their own ability following group singing. However, it is notable that most of the descriptions of cognition in the qualitative papers related to changes that occurred during the group singing sessions, or in relation to the sessions; i.e., remembering song lyrics, reminiscing prompted by the songs, and remembering/anticipating sessions. No qualitative themes appeared to reflect any changes to functional cognition outside of this context. Conversely, the quantitative studies generally focused on general changes to cognition on a neurological level [with the exception of the studies by Prickett and Moore (1991) and Moussard et al. (2014) who looked at recall and learning using song lyrics]. This highlights a potential difference in how participants describe their experience of cognition and the outcomes that researchers privilege. Dowson et al. (2019) have noted that research on music for the well-being of people with dementia is often driven by the need to quantify symptom reduction, and potentially overlooks the more nuanced benefits that participants might experience. Future research would benefit from consultation with people living with dementia to establish what aspects of cognition (or other areas) would be most meaningful to study.

In addition to measuring the impact of singing on cognition, the narrative synthesis also revealed how cognitive ability may impact engagement in singing, with some studies finding that people with lower cognitive ability were more suited to other music or non-music based activities (such as drumming or moving to music) (Clair and Bernstein, 1990; Hanson et al., 1996; Korb, 1997; Groene et al., 1998). This implies that singing may be more accessible in earlier stages of dementia progression. In contrast, qualitative studies often mentioned the accessibility of group singing, regardless of participants' abilities (Hara, 2011; McCabe et al., 2015; Unadkat et al., 2016). Although the majority of the qualitative studies featured community-based group singing programs (which implies that participants were still living at home and relatively independent), some individual accounts described participants who were in more advanced stages of dementia still participating. In an ethnographic account of a community-based singing group, Hara (2011) described how the groups were designed and facilitated to accommodate the varying needs of participants (although this was described as challenging at times). Davidson and Fedele (2011) described how formal and informal carers observed participants in later stages of dementia engaging using affect, facial expression, and body language even if they were unable to participate in the singing. This is consistent with the findings from Hanson et al. (1996) who reported a significant increase in “passive engagement” during group singing in their study. There seems to be some discrepancy between the quantitative and qualitative findings here. However, the qualitative papers did not explicitly investigate different levels of engagement across different levels of cognitive ability, and quantitative papers were generally measuring active engagement, rather than passive (with the exception of Hanson et al., 1996). Future research in this area could focus on what participants get out of being passively involved.

#### The Experience of Care-Partners

The experience of care-partners was not prominent in the quantitative/mixed-method studies, with only six studies including specific measures to assess the impact of the program for care-partners (Camic et al., 2011; Särkämö et al., 2014; Satoh et al., 2015; Lyu et al., 2018; Mittelman and Papayannopoulou, 2018; Tamplin et al., 2018), four of which were mixed methods. Conversely, only one of the qualitative papers (Dassa and Amir, 2014) did not seek perspectives from care-partners about their own experience of being involved in the groups. This is likely due to the fact that only one quantitative and four mixed method studies included caregivers (professional or familial) as part of the singing program (studies by Satoh et al., and Lyu et al., measured the impact of the program on care-partners, however, they were not part of the intervention).

The quantitative studies mostly measured outcomes related to the health and well-being of the care-partners, or their level of distress. This is unsurprising, as much of the broader literature relating to familial care-partners focuses on increasing or extending their capacity to support people with dementia to live at home (Papastavrou et al., 2007; Frankish and Horton, 2017). Two studies included measures that focused on more positive aspects of caregiving, including social support (Mittelman and Papayannopoulou, 2018), relationship quality, and positive aspects of caregiving (Tamplin et al., 2018). In the qualitative studies, the experiences of care-partners were often captured briefly, with some exceptions (Camic et al., 2011; Osman et al., 2016), as often the focus of their responses was on their perception of how the experience was for their care-partner. Nevertheless, the thematic synthesis noted specific themes related to the positive impact of group singing for care-partners in Theme 7 (“singing together supports care-partner relationships”), and subtheme 2.2 (“social support for care-partners”). The theme of flow on effects captured the way that care-partners gained knowledge and resources through the singing groups. Care-partners' experiences were also captured in the themes relating to improved psychological well-being, with several reporting the positive effects that being in the singing group had on their own mood.

Although only three quantitative studies demonstrated significant benefits for care-partners (Särkämö et al., 2014; Lyu et al., 2018; Mittelman and Papayannopoulou, 2018), the qualitative themes suggest a range of ways that care-partners may benefit from group singing programs. Favourable baseline scores and a lack of deterioration on other measures in this category suggests that there may have been some protective benefit for care-partners too (Camic et al., 2011; Satoh et al., 2015; Mittelman and Papayannopoulou, 2018; Tamplin et al., 2018), which is further supported by the qualitative findings. However, as most qualitative studies tended to focus more on the perception of the experiences of participants with dementia, future research could focus more on the specific ways that care-partners experience participation in such groups.

Several of the outcome measures used in the quantitative studies focused on negative aspects of caregiving, such as “caregiver burden,” and mental or physical ill-health. Although the financial and psychological rationale for measuring these negative features is understandable, dementia advocates have been vocal about the need to address how using such words as “burden” to describe the experiences of care-partners can increase stigma and negatively impact people with dementia. While some of the studies in this review attempted to use measures that focused on more neutral or positive elements, this was not always well-received either. Of note is a comment from a care-partner participant in the study by Clark et al. (2018), who critiqued the use of the “Positive Aspects of Caregiving Questionnaire” (PACQ), as they felt the assumption that their self-worth was linked to their role as a care-partner was offensive. This suggests that there is still some way to go in developing outcome measures that are sensitive to stigma and assumptions, and can also capture the nuance of the care-partner experience.

## Discussion

The results of this systematic-mixed-studies-synthesis highlighted some key similarities and differences in the outcomes that are reported by quantitative and qualitative studies, and the concepts they privilege measuring. An important finding from the narrative synthesis of quantitative data was that several studies reported no significant outcomes, yet observed positive responses from participants, with many studies hypothesising that good initial scores and lack of deterioration may explain this discrepancy. The results of the meta-integration revealed how participants described positive experiences and benefits that correlated with the outcomes measured in the quantitative studies. This supported the hypothesis that participants may still benefit from singing, even if they were coping relatively well prior to the intervention. These findings are important, as they support the longstanding theories that enriched environments, meaningful social engagement, and staying active can help people living with dementia to maintain their personal well-being as they progress through the stages of dementia (Kitwood, 1997; Snyder, 2006; Cridland et al., 2016; Lee K. H. et al., 2020).

The thematic synthesis also revealed some nuance around the types of benefits that participants perceive following group singing. Unadkat et al. (2016) identified a difference between in-the-moment transient benefits that participants reported (such as experiencing joy, and a positive experience), and longer-term benefits to mood that carried on after the end of each session. This distinction was also evident in the data of other qualitative studies and was acknowledged in discussion sections of several quantitative papers (Cooke et al., 2010a; Davidson and Fedele, 2011; Satoh et al., 2015). This is further supported by the findings of Pongan et al. (2019), who measured immediate impact on well-being and found significant improvements following singing and painting group interventions. In a review examining what outcomes are measured in research relating to music and dementia, Dowson et al. (2019) found that outcome measures that focus on symptom reduction were often privileged, and noted that while a focus on this may be important in the context of treatment, health economics and comparisons with previous research, the dominance of these types of measures may risk overlooking other potential benefits that music can bring. The present review found that participants and researchers observed transient or in-the-moment benefits from group singing that may not be captured by existing outcome measures. Furthermore, these transient benefits add support to the findings from Särkämö et al. (2014), who found that ongoing interventions may be necessary to maintain the benefits experienced by participants. This was also reflected in the thematic synthesis in the subtheme 1.4 (sustainability), in which participants expressed the importance of the ongoing nature of their singing groups, and in reports from the quantitative studies demonstrating similar effects for group singing and a comparable active control intervention. Future research should consider how these transient benefits and the ongoing nature of group programs may benefit people living with dementia and their care-partners. Longer-term studies are therefore needed to capture the changes and nuance that may occur over longer periods as symptoms of dementia progress and living circumstances change. A summary of key findings and recommendations can be found in Table 11.

**Table 11:** Summary of key findings and recommendations.

1. Heterogeneity of quantitative outcome measures, settings, participant demographics, and study design make it difficult to draw conclusions from the quantitative studies. High prevalence of floor and ceiling effects across the included studies suggest that future quantitative research would benefit from improved participant screening procedures. 2. Qualitative studies reveal that participants with dementia and their care-partners perceive singing to be a positive and beneficial activity. Despite the inclusion of participants with dementia, the perspectives of care-partners and professionals dominates the literature. Future research should consider strategies to enhance the inclusion of participants with dementia 3. Findings from the meta-integration suggest that benefits to well-being and quality of life may be short-term or transient; ongoing programs may be needed to maintain the benefits that singing can provide. Further research into the long-term impact of singing for people with dementia and their family care-partners is warranted.

### Limitations

There are a number of limitations to be considered in interpreting these findings. Firstly, the quality of reporting was varied, and this, combined with the heterogeneity of included studies (in design, intervention type and dosage) means that no clear conclusions can be drawn. Second, although the authors practised reflexivity throughout the synthesis process, it is possible that a priori assumptions may have influenced the grouping and coding process. Finally, while the qualitative papers did include some participants with lived experience of dementia, the perspectives of people with a diagnosis were underrepresented. Those that were included were almost all people who were attending community programs, which may be due to an assumption that people who are in later stages of dementia cannot express their opinions. Similarly, the impact on care-partners was underrepresented in the quantitative literature, while their perspectives were over-represented in the qualitative studies. The imbalance of representation for both participants with dementia and care-partners may have influenced the results of this review. Finally, this review only included articles in English.

## Conclusion

The findings of this review generally support the notion that singing, particularly in groups, may be beneficial for people living with dementia and their care-partners. Although the evidence to support specific outcomes is weak, the meta-integration of qualitative and quantitative syntheses suggests that participants in group singing may experience joy, positivity and personal well-being from being involved. Further research is required to determine the specific benefits, particularly in relation to understanding how group singing might support people in a longer-term capacity. The findings also support the view that meaningful engagement, both socially and in activities, is important for the maintenance of well-being (Kitwood, 1997; Snyder, 2006), and that such opportunities are valued by people living with dementia.

## Data Availability Statement

The original contributions presented in the study are included in the article/supplementary material, further inquiries can be directed to the corresponding author/s.

## Author Contributions

ZT was responsible for the overarching conceptualisation, design and conduct of the study, led the systematic search and review, synthesis of results, and writing of the manuscript. IC, JT, and FB conducted blind reviewing of the search results (title and abstracts, then full text reviews) and blind assessment of quality of studies, reviewed and contributed to synthesis results, and assisted in development and revision of the manuscript. IC additionally assisted in design and formatting of results tables. All authors contributed to the article and approved the submitted version.

## Funding

ZT was supported by an Australian Government Research Training Program (RTP) Stipend and RTP Fee-Offset Scholarship through the University of Melbourne. JT was funded by a National Health and Medical Research Council—Australian Research Council Grant (1106603).

## Conflict of Interest

The authors declare that the research was conducted in the absence of any commercial or financial relationships that could be construed as a potential conflict of interest.

## Publisher's Note

All claims expressed in this article are solely those of the authors and do not necessarily represent those of their affiliated organizations, or those of the publisher, the editors and the reviewers. Any product that may be evaluated in this article, or claim that may be made by its manufacturer, is not guaranteed or endorsed by the publisher.
